# GTP-Dependent Regulation of CTP Synthase: Evolving Insights into Allosteric Activation and NH_3_ Translocation

**DOI:** 10.3390/biom12050647

**Published:** 2022-04-29

**Authors:** Stephen L. Bearne, Chen-Jun Guo, Ji-Long Liu

**Affiliations:** 1 Department of Biochemistry and Molecular Biology, Dalhousie University, Halifax, NS B3H 4R2, Canada; 2 Department of Chemistry, Dalhousie University, Halifax, NS B3H 4R2, Canada; 3 School of Life Science and Technology, ShanghaiTech University, Shanghai 201210, China; guochj@shanghaitech.edu.cn; 4 Department of Physiology, Anatomy and Genetics, University of Oxford, Oxford OX1 3PT, UK

**Keywords:** CTP synthase, allostery, ammonia tunnel, guanosine-5′-triphosphate, kinetics, structure, glutaminase

## Abstract

Cytidine-5′-triphosphate (CTP) synthase (CTPS) is the class I glutamine-dependent amidotransferase (GAT) that catalyzes the last step in the de novo biosynthesis of CTP. Glutamine hydrolysis is catalyzed in the GAT domain and the liberated ammonia is transferred via an intramolecular tunnel to the synthase domain where the ATP-dependent amination of UTP occurs to form CTP. CTPS is unique among the glutamine-dependent amidotransferases, requiring an allosteric effector (GTP) to activate the GAT domain for efficient glutamine hydrolysis. Recently, the first cryo-electron microscopy structure of *Drosophila* CTPS was solved with bound ATP, UTP, and, notably, GTP, as well as the covalent adduct with 6-diazo-5-oxo-l-norleucine. This structural information, along with the numerous site-directed mutagenesis, kinetics, and structural studies conducted over the past 50 years, provide more detailed insights into the elaborate conformational changes that accompany GTP binding at the GAT domain and their contribution to catalysis. Interactions between GTP and the L2 loop, the L4 loop from an adjacent protomer, the L11 lid, and the L13 loop (or unique flexible “wing” region), induce conformational changes that promote the hydrolysis of glutamine at the GAT domain; however, direct experimental evidence on the specific mechanism by which these conformational changes facilitate catalysis at the GAT domain is still lacking. Significantly, the conformational changes induced by GTP binding also affect the assembly and maintenance of the NH_3_ tunnel. Hence, in addition to promoting glutamine hydrolysis, the allosteric effector plays an important role in coordinating the reactions catalyzed by the GAT and synthase domains of CTPS.

## 1. Introduction

**Figure 1:**
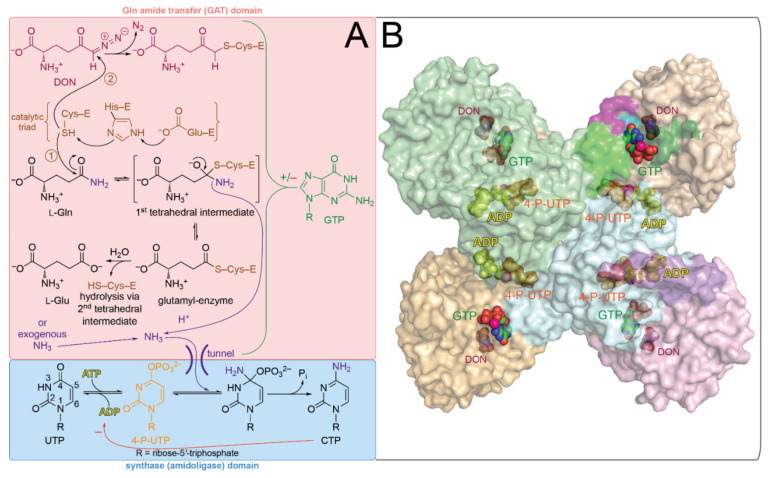
(**A**) Reactions catalyzed by CTP synthase. The active-site thiolate formed via the catalytic triad reacts with the side chain of l-Gln to form the glutamyl-enzyme (route 1). The thiolate may also react with DON, leading to covalent modification of the enzyme and inactivation (route 2). The NH_3_ generated from the hydrolysis of l-Gln is transferred to the synthase (amidoligase) domain via an intramolecular tunnel where it reacts with phosphorylated UTP (i.e., 4-P-UTP) to yield CTP (bottom). (**B**) Structure of the *Dm*CTPS tetramer with bound GTP. The tetramer is shown in surface representation with bound 4-P-UTP (orange), ADP (yellow), DON (maroon), Mg^2+^ (hot pink), and GTP (green carbon atoms) shown as spheres (PDB ID: 7DPT) [66]. The lower right protomer is colored light blue, light purple, and pink corresponding with the N-terminal synthase domain, interdomain linker, and GAT domain, respectively, as also colored in Figure 2. Those residues implicated in playing a role in GTP-dependent activation of the enzyme are shaded as shown in Figure 2 and Figure 3.

As a metabolite, cytidine-5′-triphosphate (CTP) lies at the crossroads of several major biosynthetic pathways. CTP serves as a precursor for membrane phospholipid biosynthesis [1,2,3], nucleic acid biosynthesis [4], and the glycosylation of proteins [5]. Furthermore, the radical *S*-adenosylmethionine enzyme viperin catalyzes the conversion of CTP to 3′-deoxy-3′,4′-didehydro-CTP, as part of the innate antiviral immunity [6]. CTP synthase (CTPS, EC 6.4.3.2) is the only known enzyme that catalyzes the de novo formation of the cytosine base via the conversion of uridine-5′-triphosphate (UTP) to CTP. Consequently, because of the central role of CTP in metabolism, the enzyme is recognized as a potential drug target for viral [7], protozoal [8,9,10,11,12,13,14], and *Mycobacterium tuberculosis* infections [15,16,17], as well as for cancer [4,18,19,20,21,22,23,24,25,26,27] and immunosuppression [28,29]. As a member of the class I (or triad) subfamily of glutamine-dependent amidotransferases [30], CTPS utilizes a Cys–Glu–His triad to catalyze the hydrolysis of l-glutamine (Gln) to generate nascent NH_3_ in its C-terminal Gln amide transfer (or GAT/glutaminase) domain (*Escherichia coli* CTPS (*Ec*CTPS) residues 287–544) [31,32]), which is then transferred through an intramolecular tunnel to the N-terminal synthase (or amidoligase) domain (*Ec*CTPS residues 1–266 connected to the GAT domain via an interdomain linker, residues 267–286) [32]. The lack of equilibration of the nascent NH_3_ with the solvent supported the notion of such a tunnel [33]. Upon entering the synthase domain, the nascent NH_3_ reacts at the 4-position of 4-phospho-UTP, formed through the ATP-dependent phosphorylation of UTP [34,35,36], to yield CTP (Figure 1) [37]. The enzyme can also utilize exogenous NH_3_ directly as a nitrogen source [38].

Considering the central role of CTP in metabolism, it is not surprising that CTPS is highly regulated. While the substrates ATP and UTP promote oligomerization of the enzyme from inactive monomers and dimers to active tetramers [37,39,40,41,42,43] by binding at an interfacial active site formed by the synthase domains [32,44,45], the product CTP can also induce tetramerization [43] as well as act as a feedback inhibitor [37,44]. Eukaryotic homologues of CTPS are also regulated by phosphorylation [46,47,48,49,50,51]. More recently, CTPS has been shown to form a superhelix of tetramers, and this filamentous structure (also known as cytoophidium) constitutes an additional level of regulation [52,53,54,55,56,57,58,59,60,61,62]. Finally, guanosine-5′-triphosphate (GTP) is an allosteric regulator of CTPS activity [37,63,64,65]. Indeed, among the Gln-dependent amidotransferases, CTPS is unique in that it is the only member of this family of enzymes wherein the GAT domain requires an allosteric effector for the efficient hydrolysis of Gln. Initially, GTP was believed to simply promote the Gln hydrolysis reaction; however, over the past two decades, kinetics, site-directed mutagenesis, and structural studies have revealed that the role of GTP is much more complex than originally thought. Indeed, GTP not only induces conformational changes that activate the glutaminase activity of the enzyme, but also plays a crucial role in inducing the conformational changes that permit the proper formation of the NH_3_ tunnel and synchronizing the efficient delivery of the NH_3_ from the GAT domain to the synthase domain. Herein, we review the results of site-directed mutagenesis and kinetics studies, as well as structural studies, that have led to the development of a detailed model describing the multifaceted role that GTP plays to effect the efficient catalysis of Gln-dependent CTP formation.

## 2. Regulation of CTPS Activity by GTP

### 2.1. Kinetics Studies: Laying the Groundwork

CTPS exhibits an exquisite ability to distinguish between all four ribonucleotide-5′-triphosphates in varying roles, including substrates, activators, inhibitors, and effectors of oligomerization and filament formation. Consequently, CTPS has served as a paradigm for understanding the complexities of protein regulation. Although it has been roughly 50 years since it was established that GTP serves as a positive allosteric effector of CTPS [64,65], enhancing the rate of Gln hydrolysis both in the presence and absence of substrate nucleotides, only within the last 15 years has structural information become available that provides atomic-level insights into the mechanism of allosteric activation. The first X-ray crystal structures of *Ec*CTPS with bound CTP and ADP [32,44] and *Thermus thermophilus* HB8 CTPS (*Tt*CTPS) with bound Gln [45] were reported in the mid-2000s, followed by the structures of apo-CTPS from *Sulfolobus solfataricus* (*Ss*CTPS) [67], the synthase domain of human CTPS [68], *Ec*CTPS with CTP and Gln bound [56], *Mycobacterium tuberculosis* CTPS (*Mt*CTPS) with two UTP molecules bound or UTP, AMP-PCP, and 6-diazo-5-oxo-l-norleucine (DON) bound [15], and the glutaminase domain of *Trypanosoma brucei* CTPS (*Tb*CTPS) with bound acivicin [8]. Subsequently, cryo-electron microscopy (EM) structures were reported for filaments of human CTPS [56,61] and *Drosophila melanogaster* CTPS (*Dm*CTPS) [69]. However, none of these structures contained bound GTP and, hence, insights into the details of GTP-dependent activation at the atomistic level remained elusive until very recently [66]. In the absence of structural information delineating the exact conformational changes that GTP binding effects to stimulate the glutaminase activity, site-directed mutagenesis and kinetics studies were crucial in defining the location and functional effects of GTP binding.

**Scheme 1:**
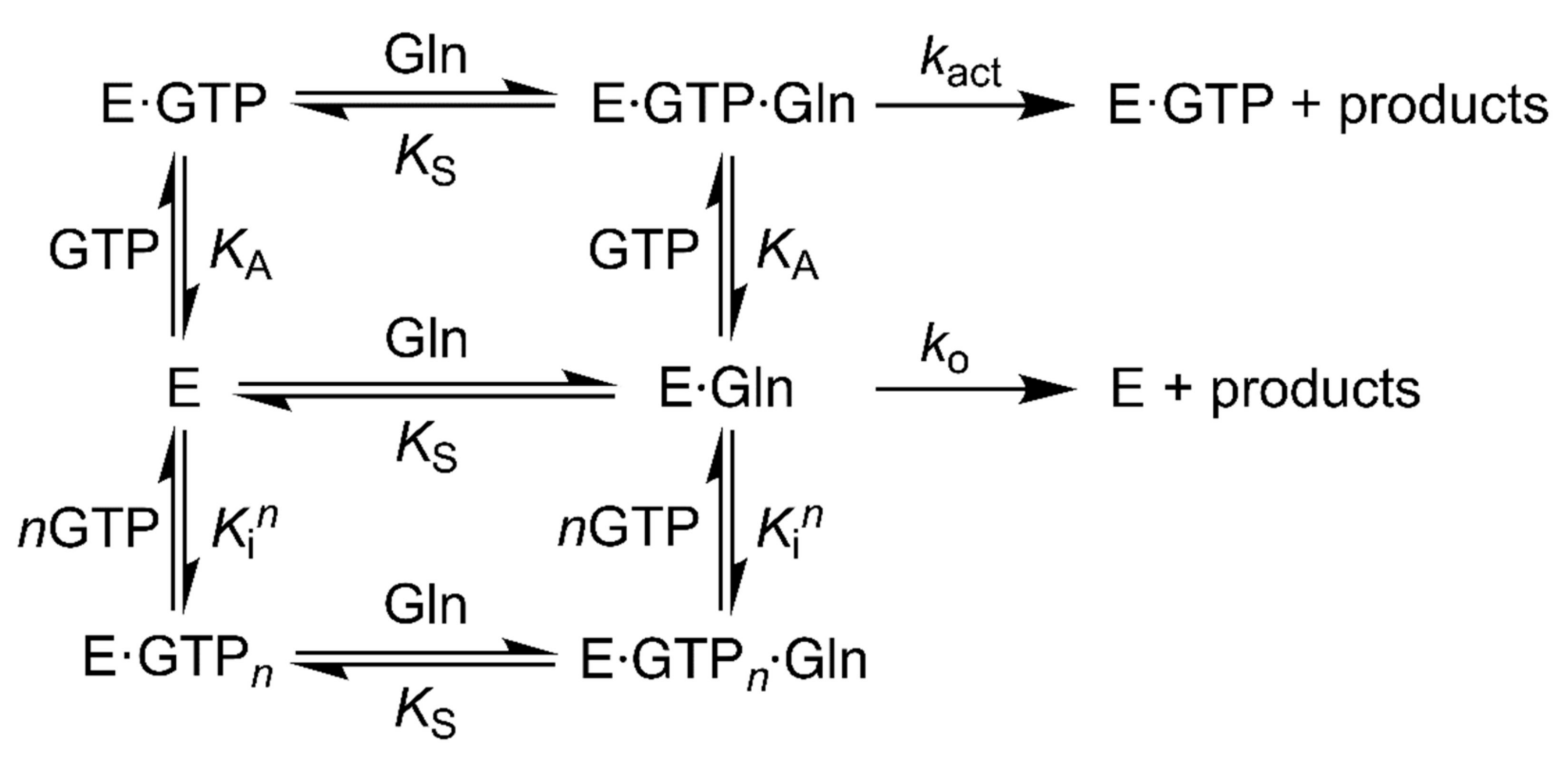
Kinetic mechanism for the activation and inhibition of CTPS by GTP. E represents the enzyme complexed with ATP and UTP. *K*_A_, *K*_i_, and *K*_S_ are apparent dissociation constants and *n* is the Hill number.

Early kinetics studies conducted by Levitzki and Koshland revealed that GTP accelerates the glutaminase activity but has little or no effect on the rate of NH_3_-dependent CTP formation [64]. Although the original kinetic model for the effects of GTP on catalysis by *Ec*CTPS appeared to be quite complex, apparently involving both negative and positive cooperativity accompanying equilibrium binding experiments [64], later studies revealed that the activation of Gln-dependent CTP formation by GTP was hyperbolic for *Ec*CTPS [70,71,72], *Lactococcus lacti* CTPS (*Ll*CTPS) [36,73], and the two yeast isozymes [74] under conditions where the enzyme was in its fully active, tetrameric state. That said, Levitzki and Koshland concluded that GTP likely exerted its effect by enhancing the rate of formation of the glutamyl-enzyme intermediate from the E·Gln Michaelis complex with little effect on the *K*_m_ value for Gln. Consistent with this hypothesis, Bearne and co-workers showed that GTP binding enhanced the inhibition of *Ec*CTPS by the intermediate/transition state analogue inhibitor glutamate γ-semialdehyde, thereby suggesting that GTP promotes the glutaminase activity by stabilizing a protein conformation that binds the tetrahedral intermediate(s) formed during Gln hydrolysis [75]. Similar results were obtained by Willemoës with *Ll*CTPS [76]. The notion that the allosteric binding of GTP causes subtle conformation changes and re-organization of the environment at the oxyanion hole to facilitate the glutaminase reaction was consistent with the behavior of other amidotransferases [77,78,79]. Later studies revealed that at elevated concentrations, GTP behaved as a weak negative allosteric effector of *Ec*CTPS inhibiting Gln-dependent CTP formation [71]. Interestingly, GTP did not inhibit the enzyme’s intrinsic glutaminase activity. Furthermore, GTP inhibited NH_3_-dependent CTP formation. Overall, these and other kinetics studies on the activation and inhibition of CTPS by GTP have been interpreted in terms of kinetic mechanisms similar to the one shown in Scheme 1, which yields the initial velocity Equation (1), where *k*_o_ and *k*_act_ are the rate constants for the formation of CTP in the absence and presence of GTP, respectively, *K*_A_ is the dissociation constant for the E·GTP complex, *K*_i_*^n^* is the apparent dissociation constant for the non-productive E·(GTP)*_n_* complex, and *n* is the Hill number [71,72].

(1) $$\frac{v_{i}}{{\left[E\right]}_{T}}=\frac{\left(k_{o}+\left(\frac{k_{\mathrm{act}}\left[\mathrm{GTP}\right]}{K_{A}}\right)\right)\left[\mathrm{Gln}\right]}{\left(1+\frac{\left[\mathrm{GTP}\right]}{K_{A}}+{\left(\frac{\left[\mathrm{GTP}\right]}{K_{i}}\right)}^{n}\right)\left(K_{S}+\left[\mathrm{Gln}\right]\right)}$$

Typically, saturating concentrations of Gln are employed so that Equation (1) reduces to Equation (2) to simplify the kinetic analysis.

(2) $$\frac{v_{i}}{{\left[E\right]}_{T}}=\frac{k_{o}+\left(\frac{k_{\mathrm{act}}\left[\mathrm{GTP}\right]}{K_{A}}\right)}{1+\frac{\left[\mathrm{GTP}\right]}{K_{A}}+{\left(\frac{\left[\mathrm{GTP}\right]}{K_{i}}\right)}^{n}}$$

Variations of Equation (1) have also been utilized such as Equation (3) [36,73,76].

(3) $$\frac{v_{i}}{{\left[E\right]}_{T}}=k_{\mathrm{cat},1}+\frac{k_{\mathrm{cat},2}\left[\mathrm{GTP}\right]}{K_{A}+\left[\mathrm{GTP}\right]}$$

The observation that GTP could inhibit the utilization of exogenous NH_3_ in a concentration-dependent manner suggested that the binding of GTP could block the access of NH_3_ to the enzyme and that the entry point for the exogenous NH_3_ was located in the GAT domain. While GTP weakly inhibits NH_3_-dependent CTP formation by *Ec*CTPS at all concentrations [71], the inhibition is more pronounced when the active-site Cys is covalently modified by 6-diazo-5-oxo-l-norleucine (DON, Figure 1) [64], which mimics the glutamyl-enzyme intermediate, or when the enzyme is inhibited by glutamate γ-semialdehyde, which mimics the tetrahedral intermediate(s) formed during hydrolysis [75]. It is possible that the negative cooperativity reported by Levitski and Koshland [64] reflected the conformational changes that accompany the inhibition of both the NH_3_- and Gln-dependent CTP formation at elevated concentrations of GTP (vide infra) [71,72].

Similar to *Ec*CTPS, GTP binding inhibited NH_3_-dependent CTP formation by DON-inactivated *Ll*CTPS, although, in this case, the inhibition by GTP exhibited negative cooperativity (*K*_i_ = 0.40 mM, *n* = 0.39) [36]. Furthermore, GTP did not inhibit NH_3_-dependent CTP formation catalyzed by *Ll*CTPS.

In addition to these differences, *Ec*CTPS and *Ll*CTPS exhibited differences in how Gln hydrolysis was regulated by GTP. While Levitzki and Koshland [64] found that *Ec*CTPS catalyzed the GTP-activated hydrolysis of Gln in the presence of UTP and ADPNP at a steady-state rate similar to the rate of the GTP-activated synthesis of CTP and with a similar degree of enhancement in the presence of GTP, Willemoës and Sigurskjold [36] found that GTP-dependent activation of the *Ll*CTPS-catalyzed glutaminase reaction never attained the same levels as the overall reaction for GTP-activated Gln-dependent CTP formation, including in the presence of UTP and the non-hydrolyzable ATP analogue adenosine-5′-[γ-thio]triphosphate (ATPγS). Unlike *Ec*CTPS, the affinity of *Ll*CTPS for GTP was increased in the presence of ATP and UTP. Indeed, the degree of saturation with ATP and UTP, unlike ATPγS and UTP, had a pronounced influence on the *K*_A_ value for GTP binding, with the value of *K*_A_ decreasing with lower concentrations of the substrate nucleotides [73]. Moreover, at low GTP concentrations ranging from ~10 to 100 µM, the rate of Gln hydrolysis exceeded that of CTP synthesis, resulting in uncoupling of the two reactions. However, as the concentration of GTP was increased, GTP either coordinated or coupled the two reactions [36]. In addition, the inhibition typically observed for GTP at concentrations above 1 mM was relieved in the presence of ATPγS and UTP. Overall, Willemoës and Sigurskjold [36] concluded that while the rate of the glutaminase reaction of *Ec*CTPS is independent of the UTP-phosphorylation reaction, for *Ll*CTPS, the 4-phosphorylated UTP intermediate appears to act as a weak activator of Gln hydrolysis, which, in a synergistic relationship, is greatly enhanced by the allosteric binding of GTP with concomitant enhancement of the enzyme’s binding affinity for GTP. Thus, GTP binding serves to coordinate the phosphorylation of UTP with Gln hydrolysis to promote efficient CTP synthesis by *Ll*CTPS.

### 2.2. Structure-Activity Studies

Lunn et al. [72] examined the ability of a variety of GTP analogues to act as allosteric activators of *Ec*CTPS. The structural requirements for activation proved to be quite stringent, with only a few analogues of GTP capable of activating Gln-dependent CTP formation: GTP ≈ 6-thio-GTP > inosine-5′-triphosphate ≈ guanosine-5′-tetraphosphate > *O*^6^-methyl-GTP > 2′-deoxy-GTP. Overall, the 5′-triphosphate, 2′-OH, and 3′-OH groups were required for full activation, while the 2-NH_2_ group was required for binding recognition, and substituents at the 6-position of the purine ring played an important role in activation. On the other hand, the structural requirements for inhibition appeared to be quite lax, with all GTP analogues able to weakly inhibit the utilization of exogenous NH_3_ as a substrate by the enzyme. Nucleotide and nucleoside analogues of GTP and guanosine, respectively, inhibited both NH_3_- and Gln-dependent CTP formation to a similar extent. The inhibition appeared to be due solely to the purine base and, with the exceptions of inosine, ITP, and adenosine, was insensitive to the identity of the purine. 8-Oxoguanosine (IC_50_ = 80 µM) was initially identified as a good inhibitor, binding 4-fold tighter than guanosine [72]. Subsequent studies supported the notion that only the purine heterocycle was required for inhibition, with xanthines and uric acids also exhibiting inhibition [80]. For example, 1,3,7-trimethyluric acid was identified as a particularly good inhibitor of *Ec*CTPS-catalyzed NH_3_- and Gln-dependent CTP synthesis (IC_50_ = 70 µM). Indeed, when this inhibition strategy was applied to CTPS from *T. brucei*, uric acid (IC_50_ = 100 µM) was shown to be a reasonably effective inhibitor of that enzyme [14]. Overall, these observations suggested that the bound purine base was sufficient to inhibit the use of exogenous NH_3_ and weakly impede GTP binding and activation.

### 2.3. Site-Directed Mutagenesis and Kinetics Studies: Mapping Regions of CTPS Contributing to GTP-Dependent Effects

Kinetics studies have provided invaluable insights into the role of GTP as an allosteric activator and have implicated several specific residues and regions of CTPS that participate in GTP-dependent activation of the enzyme. For the following discussion, the nomenclature used for structural elements (i.e., loops and lids) is that introduced by Baldwin and co-workers for *Ec*CTPS [32].

#### 2.3.1. The L13 Loop: Limited Proteolysis

**Figure 2:**
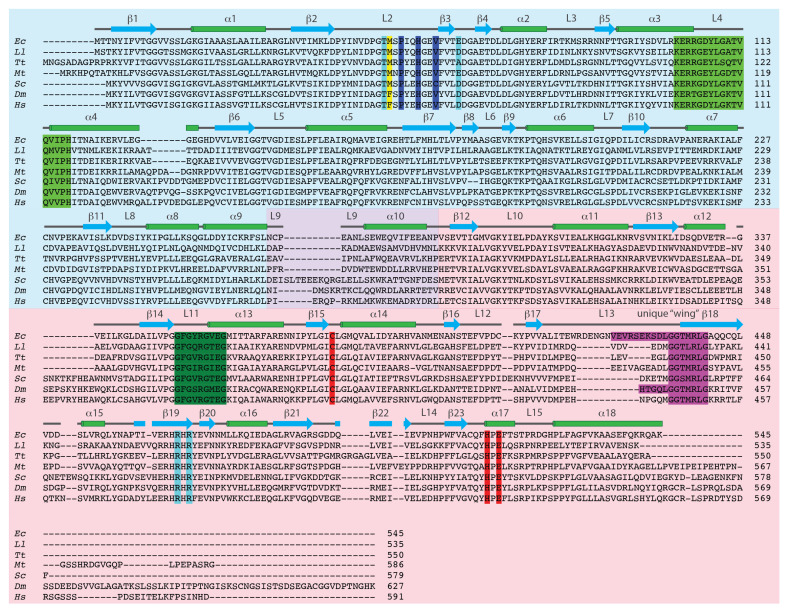
Amino acid sequence alignment of selected CTP synthase proteins. Regions of the primary amino acid sequence corresponding to the N-terminal synthase (amidoligase) domain, the interdomain linker, and the C-terminal GAT domain are shaded light blue, light purple, and pink, respectively. Residues comprising the Cys–Glu–His triad are highlighted in red and the residues comprising the gate at the distal end of the NH_3_ tunnel are highlighted in dark blue. Residues highlighted in light green, dark green, cyan, and purple play a role in GTP-dependent activation and correspond to the similarly colored residues in the structure shown in Figure 3A. The secondary structural assignments are those proposed by Baldwin and co-workers for *Ec*CTPS [32,44]. The species include: *Escherichia coli* (*Ec*, P0A7E5), *Lactococcus lacti* (*Ll*, O87761), *Thermus thermophilus* (*Tt*, Q72IN0), *Mycobacterium tuberculosis* (*Mt*, P9WHK7), *Saccharomyces cerevisiae* (*Sc*, P28274, URA7), *Drosophila melanogaster* (*Dm*, Q9VUL1), and *Homo sapiens* (*Hs*, P17812, CTPS1).

Using trypsin-catalyzed proteolysis of *Ec*CTPS, Bearne and co-workers showed that Arg 429 and Lys 432 (Table 1) reside on an exposed loop (L13, Figure 2) [81]. Subsequent site-directed mutagenesis studies revealed that R429A *Ec*CTPS exhibited wild-type levels of NH_3_-dependent CTP formation; however, the values of *k*_cat_ and *k*_cat_/*K*_m_ for Gln-dependent CTP formation were reduced ~10- and ~20-fold, respectively, relative to the wild-type enzyme. The *K*_m_ for Gln was not significantly altered. Activation of the glutaminase activity was reduced 6-fold at saturating concentrations of GTP, and the GTP-binding affinity was reduced 10-fold. These observations indicated that Arg 429 plays a role in both GTP-dependent allosteric activation of Gln hydrolysis and GTP binding [81].

Arg 429 and Lys 432 lie close to a conserved sequence motif [GG(TS)(ML)RLG)] (shaded purple in Figure 2) within the GAT domain that Willemoës suggested played a role in GTP-dependent activation [85]. This suggestion was based on the observation that the motif was only present in CTPSs and not in the GAT domain of other class I Gln-dependent amidotransferases, which are not regulated by GTP binding. Through site-directed mutagenesis and kinetics studies, Willemoës demonstrated that Thr 431 and Arg 433 within this motif of *Ll*CTPS (Thr 438 and Arg 440 in *Ec*CTPS) play a role in GTP-dependent activation of Gln hydrolysis [85]. Since the T431V *Ll*CTPS variant exhibited wild-type affinity for GTP, it was concluded that the hydroxyl group of Thr 431 plays a role only in the structural changes accompanying GTP-dependent activation. However, Willemoës did observe a 10–17-fold decrease in the GTP-binding affinity with the R433A *Ll*CTPS variant, similar to the 10-fold reduction in GTP-binding affinity observed for the R429A *Ec*CTPS variant. Hence, it appears that the conserved sequence motif and adjacent residues of the L13 loop are involved in both GTP binding and activation.

#### 2.3.2. The L4 Loop

Iyengar and Bearne [82] conducted scanning alanine mutagenesis on a highly conserved region between residues 102 and 118 in *Ec*CTPS (shaded light green in Figure 2) and showed that residues located centrally within this region functioned to ensure efficient coupling of Gln-dependent NH_3_ formation to the synthase activity. Significantly, the D107A and L109A variants (Table 1) exhibited wild-type levels of Gln hydrolysis and NH_3_-dependent CTP formation; however, Gln-dependent CTP formation was markedly impaired. Interestingly, *K*_A_ for GTP binding, the GTP-dependent glutaminase activity, the *K*_m_ for Gln, and UTP- and ATP-dependent tetramerization were not altered significantly by the amino acid substitutions. These observations suggested that the D107A and L109A substitutions altered the normal structure of the NH_3_ tunnel (i.e., the tunnel is leaky or constricted) or caused a structural perturbation that prevented the formation of a functioning tunnel, the effect being more pronounced for the L109A variant [82]. To test the hypothesis that the L109A substitution was in some way constricting the NH_3_ tunnel, Lunn and Bearne [83] compared the kinetic parameters of Gln and NH_3_ with those of the corresponding bulkier substrates l-γ-glutamyl hydroxamate (Gln-OH) and NH_2_OH, as well as the coupling ratios (Equations (4) and (5)) observed for Gln and Gln-OH with the wild-type and L109A *Ec*CTPSs. Interestingly, the L109A variant exhibited greater uncoupling with the bulkier nascent NH_2_OH, derived from Gln-OH hydrolysis, than with NH_3_ derived from Gln hydrolysis. These observations were consistent with the L109A variant possessing a constricted or malformed NH_3_ tunnel.

(4) $$\mathrm{sub}-\mathrm{saturating}\;\mathrm{couling}\;\mathrm{ratio}=\frac{{\left(k_{\mathrm{cat}}/K_{m}\right)}_{\mathrm{CTP}\;\mathrm{formation}}}{{\left(k_{\mathrm{cat}}/K_{m}\right)}_{\mathrm{glutaminase}\;\mathrm{activity}}}$$

(5) $$\mathrm{saturating}\;\mathrm{couling}\;\mathrm{ratio}=\frac{{\left(k_{\mathrm{cat}}\right)}_{\mathrm{CTP}\;\mathrm{formation}}}{{\left(k_{\mathrm{cat}}\right)}_{\mathrm{glutaminase}\;\mathrm{activity}}}$$

These authors also examined the kinetic properties of the L109F variant to see if the NH_3_ tunnel could be fully blocked. This substitution, however, yielded a marked reduction in the glutaminase activity arising from either a local perturbation at the Gln site such that Gln binding was not affected but its rate of hydrolysis was reduced, or a failure of GTP binding to induce the appropriate conformational change that promotes hydrolysis of Gln. The authors favored the latter scenario since the *K*_A_ value for GTP binding was similar to that of the L109A variant, indicating that the loss of glutaminase activity did not arise from impaired binding of GTP. A similar kinetic profile for the R105A variant also suggested that Arg 105 was required for efficient Gln turnover [82].

#### 2.3.3. The Lid L11 Loop

Based on the structures of *Ec*CTPS (vide infra) [32] and *Tt*CTPS [45], Willemoës and co-workers identified a loop region denoted as lid L11 (residues 354–362 of *Ec*CTPS, shaded dark green in Figure 2) [32], which immediately preceded the amino acid residues comprising the oxyanion hole as a potential structural motif involved in the GTP-dependent activation of CTPS [84]. Indeed, some of the residues of lid L11 were not detected in electron density maps [45], indicating that this loop is highly flexible. The R359M, R359P, and G360P *Ll*CTPS variants (R356 and G357 in *Ec*CTPS) exhibited 10- to 50-fold reductions in GTP-dependent activation of Gln-dependent CTP formation accompanied by 4- to 10-fold increases in the *K*_A_ for GTP (Table 1). While the uncoupled glutaminase activities for these variants were not activated by GTP, the G360A variant was about 2-fold more active than wild-type *Ll*CTPS [84]. Overall, these observations suggested that interaction of GTP with the lid L11 is required for the activation of Gln-dependent CTP synthesis. Indeed, deletion of the charge of the side chain in the R359M *Ll*CTPS variant supported the notion put forward by Baldwin and co-workers [32] that Arg 359 interacts with GTP and may act as a lever to alter the conformation of the lid L11, leading to a conformation favoring Gln hydrolysis [84].

Interestingly, one of the first site-directed mutagenesis studies to probe the role of residues in the GAT domain in the region of the lid L11 was conducted by Weng and Zalkin [31]. These authors altered Gly 352 in the *Ec*CTPS GAT domain, showing that the G352P variant (Table 1) exhibited no detectable Gln-dependent CTP formation but was almost fully active when NH_4_Cl was the ammonia source. Furthermore, the catalytic Cys 379 at the active site could not be labeled by DON. Although these authors suggested that the mutation either prevented formation of the covalent glutamyl-enzyme intermediate or disrupted NH_3_ transfer, their results did not preclude the possibility that GTP-dependent activation was disrupted. Only later did the structure of *Ec*CTPS reveal that Gly 352 resides at the junction between the rigid portion of the oxyanion hole and the lid L11 loop. Substitution of Gly by Pro at position 352 likely abrogated the activation of the glutaminase activity by hindering the flexibility of the lid L11 region [84].

#### 2.3.4. “Pinching” the NH_3_ Tunnel

The X-ray crystal structures of *Ec*CTPS (with no GTP bound, vide infra) revealed that the NH_3_ tunnel had a constriction formed by three residues (Pro 54, His 57, and Val 60, shaded dark blue in Figure 2) at the entryway to the synthase domain that are highly conserved among CTPSs [32,44]. To permit the passage of NH_3_, which has a molecular diameter close to 4 Å, the constriction must open since it is only~2.4 Å in diameter in the crystal structures. The proximity of this putative molecular gate to the proposed GTP-binding site suggested that GTP might play a role in controlling the opening of the gate to regulate the delivery of NH_3_ to the synthase domain. By conducting kinetics and biophysical analyses on *Ec*CTPS variants with different amino acid substitutions of the gate residue Val 60, which resides at the most constricted part of the NH_3_ tunnel, McCluskey and Bearne [86] explored the role of the putative NH_3_ gate in coordinating the reactions required for CTPS catalysis. All amino acid substitutions (i.e., Ala, Cys, Asp, Trp, and Phe) at position 60 appeared to cause local structural perturbations that had a detrimental effect on the ability of GTP to bind and activate the enzyme, as anticipated based on the proximity of Val 60 to the GTP-binding site [32,82,83,84]. Most interestingly, these authors discovered that replacement of Val 60 by the bulkier Phe residue unveiled the coordinated role of both GTP binding and the glutaminase activity in facilitating the passage of NH_3_ through the tunnel gate. The V60F variant exhibited a slightly reduced coupling efficiency at maximal glutaminase activity that was ameliorated by slowing the rate of Gln hydrolysis, suggesting a “bottleneck” effect. Moreover, the inability of V60F *Ec*CTPS to use exogenous NH_3_ as a substrate could be overcome in the presence of GTP, and more so if the enzyme was modified by DON. When NH_2_OH was employed as an alternative, bulkier substrate, its use was more efficient when it was concomitant with the glutaminase reaction. Hence, GTP-dependent activation appears to act in concert with the glutaminase activity to open the molecular gate for the passage of NH_3_ [86].

### 2.4. Regulatory Effects of NADH

Baldwin, Kollman, Gitai, and co-workers observed that nicotinamide-containing compounds were modest inhibitors of *Ec*CTPS with IC_50_ values following the trend: 1-methyl 1,4-dihydronicotinamide (IC_50_ = 140 µM) < NADH ≈ NADPH << NADP^+^ < NAD^+^ << 1-methyl nicotinamide (IC_50_~4000 µM), indicating that the reduced nicotinamide ring is sufficient for inhibition [87]. NADH inhibition was enhanced in the presence of increasing concentrations of GTP, including those concentrations for which GTP is either activating or inhibitory. Correspondingly, in the presence of a fixed concentration of NADH, the dependence of the activating and inhibitory effects of GTP were more acute (i.e., lowering both the EC_50_ and IC_50_ values for activation and inhibition, respectively). Hence, NADH enhanced the allosteric regulation of *Ec*CTPS by GTP in a mutual fashion. Furthermore, these authors found that increasing the concentration of GTP enhanced the inhibition by CTP; conversely, in the presence of CTP, the EC_50_ and IC_50_ values for GTP were reduced. The observations that NADH, CTP, and GTP mutually enhanced each other’s effects suggested that they interact with a common intermediate enzyme state. Finally, Barry et al. [53] demonstrated that CTP was a more effective inhibitor under conditions that favored filament formation by the enzyme (i.e., elevated concentrations of CTP or the enzyme). Using the E227R *Ec*CTPS variant, which obviates the ability of the enzyme to form filaments and reduces the inhibitory effects of CTP, Baldwin, Kollman, Gitai, and co-workers observed that the EC_50_ and IC_50_ values were shifted to higher values for GTP-dependent activation and inhibition, respectively [87]. This intriguing observation suggested that GTP interacted with the inhibitory filament, which was an insightful claim, considering that at that point, GTP had not been observed in any of the high-resolution cryo-EM reconstructions of CTPS filaments [87]. Although NADH did not appear to bind at the UTP- and CTP-binding sites, it remains unclear where NADH binds to exert its effects on the GTP-dependent activity of *Ec*CTPS [44,87]. Overall, these observations highlight the important regulatory role that GTP plays by inducing conformational changes that affect the binding and activity of other nucleotide ligands, which is also consistent with the notion that the 4-phospho-UTP and GTP could act as coactivators of the glutaminase reaction as pointed out by Willemoës [36].

## 3. Structural Studies Intimating the Location of the GTP-Binding Site and the Role of GTP-Dependent Activation in NH_3_ Translocation

**Figure 3:**
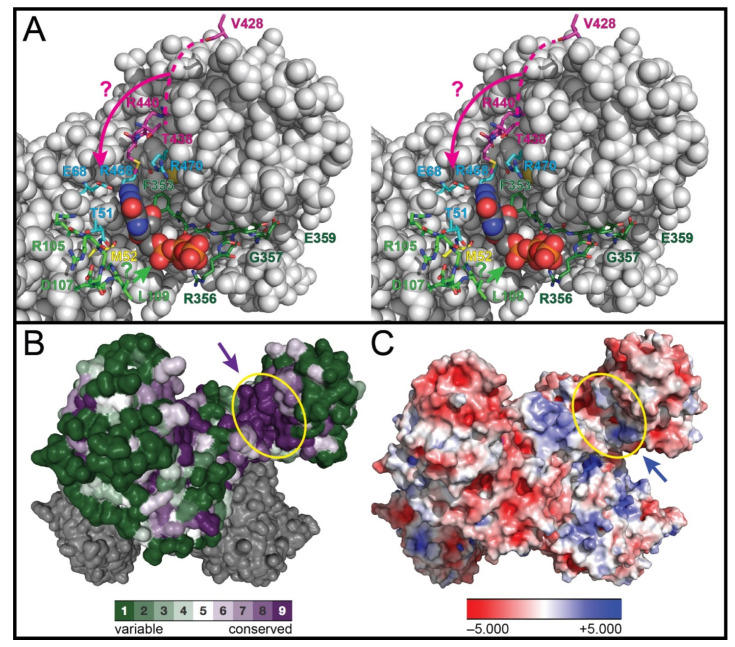
Conservation, electrostatics, and interacting residues of the GTP-binding site. (**A**) Stereoview of the GTP-binding site (sphere representation) is shown with the same orientation as in panels B and C. GTP (space-filling representation) is shown as modeled into the cleft of *Ec*CTPS (PDB ID: 1S1M) by Baldwin and co-workers [32,44] with those residues implicated in GTP-dependent activation of CTPS (Table 1) highlighted: residues of the 104–110 loop (light green; especially Asp 107 and Leu 109), residues 352–360 of the L11 lid (dark green; especially Gly 352, Arg 356, and Gly 360), residues of the 429–440 loop (purple; especially Arg 429, possibly Lys 432, Thr 438, and Arg 440), residues Glu 68, Arg 468, Arg 470, and Thr 51 (cyan), and Met 52 (yellow). The coloring of the amino acid residues corresponds to the shading of the amino acids in the sequence alignment shown in Figure 2. The light green and magenta arrows suggest movement of the similarly colored residues upon GTP binding. Clearly, the enzyme must undergo a change in conformation (suggested by arrows) from that shown so that the protein interacts with the 6-position of the purine ring since GTP-dependent activation of Gln-dependent CTP formation requires the presence of a carbonyl group at the 6-position of GTP. (**B**) Conservation of residues based on a multiple sequence alignment prepared and rendered in surface representation using the CONSURF server [88] is shown for two chains of the CTPS tetramer. Highly conserved residues (purple) line the GTP-binding cleft denoted by the purple arrow. (**C**) Electrostatic potential surface of the CTPS tetramer (surface representation) is shown with the same orientation as in panel B. The blue arrow denotes the electropositive region that binds the 5′-triphosphate moiety of GTP.

A breakthrough in the study of CTPS occurred when the first X-ray crystal structures of CTPS were reported in 2004. Baldwin and co-workers reported the structure of *Ec*CTPS in its ligand-free form [32] and Hirotsu and co-workers reported the structure of *Tt*CTPS with bound Gln [45]. A year later, Baldwin’s laboratory reported the structure of the *Ec*CTPS·ADP·CTP product complex [44]. These seminal structures helped to delineate the spatial arrangement of the site of Gln hydrolysis, the NH_3_ tunnel, the binding of nucleotides at the synthase site, and the protein oligomerization interfaces, as well as inform the design and interpretation of site-directed mutagenesis studies. Unfortunately, no electron density for GTP was present, despite GTP being present in the crystallization solution for the *Ec*CTPS structures [44,87]. Hence, few direct insights were provided regarding the conformational changes accompanying GTP binding. However, by comparing the GAT domain with structurally related GTP-binding proteins (e.g., EF-Tu and EF-G), Baldwin and co-workers [32] identified a potential binding site for GTP located adjacent to the entrance to the glutaminase active site in a deep cleft residing between the GAT and synthase domains close to the A–A’ dimer interface (Figure 3). The cleft is formed by residues 50–55, 297–301, 353–356, 438–441, 468, and 470. Baldwin and co-workers constructed a model of GTP bound at this site (Figure 3A) [32], which has permitted the results from site-directed mutagenesis and kinetics experiments to be put into context. Indeed, this binding site has now been confirmed with the first structure of CTPS with GTP bound (vide infra) [66].

The GTP-binding site resides in a region of highly conserved amino acid sequence (Figure 3B), which exhibits a predominantly negative electrostatic potential surface for interacting with the 5′-triphosphate moiety of GTP (Figure 3C). With the exception of the amino acid residues on the flexible loop L13 between Val 428 and Thr 438, all the conserved residues listed in Table 1 that were implicated in the GTP-dependent activation of Gln-dependent CTP formation by site-directed mutagenesis experiments line this cleft (Figure 3A). In the structure, loop L4 (*Ec*CTPS residues 105–114) resides between the GAT and synthase active sites and forms part of the rim of the GTP-binding site. Arg 468, Arg 470, and Glu 68 appear to interact with the purine moiety of GTP [32]. Significantly, Leu 109 is located on a flexible loop of an adjacent subunit, which extends over the deep cleft separating the GAT and synthase sites. This places Leu 109 above the opening that Endrizzi et al. [32] had identified as a putative entry point for NH_3_ to access the solvent-filled “vestibule” connecting the GAT active site and the GAT/synthase interface. Leu 109 is not interacting with GTP in the model. However, the kinetic properties of the L109A *Ec*CTPS variant suggested that the residue is involved with GTP-dependent activation of the enzyme. Clearly, Leu 109 does not directly cause a constriction in the NH_3_ tunnel, but substitution by Ala alters the kinetics by interfering with the appropriate conformational changes that accompany GTP binding and proper formation of the NH_3_ tunnel. As such, the resulting tunnel may still be constricted or malformed. Baldwin and co-workers suggested that a polypeptide linker connecting the L4 loop and the symmetry-related subunit could mediate coupling between ATP-binding and Gln hydrolysis [44]. Most interestingly, the loop L4 residues Arg 105 and Leu 109 [82], as well as the loop L13 residue Arg 429 [81], do not make any key contacts with other residues in the apo-*Ec*CTPS structure, consistent with the numerous studies that suggest that GTP binding induces substantial conformational changes in *Ec*CTPS [34,64,71,75,89] and *Ll*CTPS [36]. Such changes may serve to appropriately reposition the triad residues His 515 and Glu 517 for catalysis and open the NH_3_ tunnel [86].

Baldwin and co-workers also noted that a 3-Å wide entrance to a solvent-filled “vestibule” that connects the GAT active site and the GAT/synthase interface is present at the base of the GTP-binding cleft [32]. That GTP binds and occludes this entryway for exogenous NH_3_ was supported by the inhibition of NH_3_-dependent CTP formation by elevated concentrations of GTP [71,72,75]. Furthermore, GTP could serve as a “bung” to prevent the loss of nascent NH_3_ and ensure the fidelity of NH_3_ transfer to the synthase domain.

Analysis of the X-ray structure of *Tt*CTPS [45] suggested that the binding of ATP and UTP induce a conformational change that brings the GAT and synthase domains closer together, permitting formation of the NH_3_ tunnel. Indeed, the various residues that have been implicated in GTP-dependent activation of the glutaminase activity (especially L109A in *Ec*CTPS) are brought closer together in the structure. This observation suggests that an even more prominent structural transition could be associated with GTP binding. Interestingly, Hirotsu and co-workers did not observe the presence of an NH_3_ tunnel in the structures of *Tt*CTPS or the *Tt*CTPS·3SO_4_^2−^ and *Tt*CTPS·Gln complexes, leading them to conclude that *Tt*CTPS must undergo an extensive conformational change upon binding ATP and UTP [45]. Similarly, a patent NH_3_ tunnel was not observed in the structure of *Mt*CTPS [15]. Based on the site-directed mutagenesis and kinetics studies outlined above and sequence conservation, Hirotsu and co-workers [45] identified two consensus sequences associated with GTP binding and activation: residues 111–130 (residues 102–121 in *Ec*CTPS, partly shaded light green in Figure 2) in the synthase domain and residues 438–444 (residues 436–442 in *Ec*CTPS, shaded purple in Figure 2) in the GAT domain. These two consensus sequences are located 15–25 Å apart in the *Tt*CTPS structure, and changes in conformation upon the binding of ATP and UTP were suggested to move these sequences closer together to form the GTP-binding site (arrows, Figure 3A).

## 4. GTP-Dependent Activation of CTPSs from Other Species

While the GTP-dependent activation of *Ec*CTPS and *Ll*CTPS have been studied in the most detail as outlined above, the activation of Gln-dependent CTP formation by GTP has either been noted or partially characterized in a variety of other organisms (Table 2).

### 4.1. Thermus thermophilus

Although structural information is available for *Tt*CTPS, only limited kinetics studies have been conducted with the enzyme, revealing that it is activated by GTP with a Hill number of 2.1 [45].

### 4.2. Chlamydia trachomatis

Limited kinetics studies revealed that *Ct*CTPS is activated by GTP with a *K*_A_ value of 7 µM [92].

### 4.3. Trypanosoma brucei

Fijolek et al. [9] found that GTP activated Gln-dependent CTP formation catalyzed by *Tb*CTPS with a *K*_A_ value of 70 µM. Steeves and Bearne [14] also observed activation of *Tb*CTPS by GTP, but inhibition at concentrations above 0.2 mM, with values of *k*_o_, *k*_act_, *K*_A_, *K*_i_, and *n* equal to 0.16 s^−1^, 0.87 s^−1^, 57 µM, 272 µM, and 4.2, respectively. Similar to *Ec*CTPS, purine derivatives such as GTP, guanosine, caffeine, and uric acid inhibited *Tb*CTPS with IC_50_ values of 460, 380, 480, and 100 µM, respectively.

### 4.4. Giardia intestinalis

*Gi*CTPS from the intestinal protozoan parasite *G. intestinalis* is activated by GTP (*K*_A_ = 60 µM), although, whether the requirement for GTP was absolute or not was not established [94]. Additionally, GDP (*K*_A_ = 0.10 mM) and dGTP (*K*_A_ = 0.23 mM) were found to activate the enzyme but not to the same degree as GTP (GTP > GDP > dGTP). The concentration of GTP did not affect the apparent dissociation constants for ATP, UTP, or Gln. Interestingly, the amino acid sequence of *Gi*CTPS contains two inserts in the synthase domain and one in the GAT domain that are not present in the yeast, human, and *E. coli* enzymes [12]. The role of these additional amino acid inserts is unknown.

### 4.5. Plasmodium falciparum

Curiously, *Pf*CTPS from the human parasite *P. falciparum* contains two inserted stretches of 42 and 233 amino acids within the GAT domain, suggesting that this novel difference from other CTPSs might be capitalized on for drug development [10]. The gene encoding *Pf*CTPS containing partial synthetic sequences substituted with preferred codons utilized by *E. coli* has been expressed heterologously in *E. coli* and the resulting enzyme shown to be active [93]. Although GTP was claimed to activate *Pf*CTPS, no data were reported.

### 4.6. Saccharomyces cerevisiae

There are two isoforms of CTPS in *S. cerevisiae* encoded by the *URA7* and *URA8* genes [106,107]. For *Sc*CTPS7 encoded by *URA7*, which is responsible for the majority of CTP made in vivo [107], activation by GTP was not found to be an absolute requirement for CTPS activity but did enhance the activity 4-fold [96]. Double-reciprocal plots of the initial velocity data, obtained with Gln as the variable substrate with various fixed concentrations of GTP, exhibited concave-downward curves, consistent with negative cooperative kinetics with respect to Gln and GTP. Carman and co-workers conducted a series of studies examining the phosphorylation of *Sc*CTPS7 by protein kinases A (PKA) and C (PKC) [47,97,98,99,108]. Phosphorylation by PKA at Ser 424 [58] in the GAT domain [99] resulted in a slight decrease in the *K*_A_ value for GTP from 15.3 to 12.0 µM, but did not have a significant effect on the apparent *V*_max_ and *K*_m_ values with respect to Gln when measured either in the absence or presence of GTP. Interestingly, the phosphorylation did eliminate the negative cooperativity exhibited with respect to Gln [98]. This behavior was also observed when the enzyme was phosphorylated by PKC, including a slight decrease in the *K*_A_ value from 15.7 to 12.2 µM [97]. Finally, to assess the role of the mobile L11 loop adjacent to the allosteric GTP-binding cleft in filament formation by *Sc*CTPS7, the abilities of the GFP-tagged variants R381M, R381P, and G382A (*cf*. Arg 356 and Gly 357 in *Ec*CTPS) to form filaments in vivo were examined. Interestingly, these variants exhibited a ~3.1-fold increase in the number of yeast cells forming filaments compared with strains expressing wild-type *Sc*CTPS7. Moreover, the median length of the filaments was increased by 33% and 15% for the R381M and R381P variants, respectively, relative to the wild-type enzyme. The G382A variant also exhibited an increased number of cells with filaments; however, the median length of the filaments was reduced by 25%. To date, this is the only report of residues at the GTP-binding site contributing to regulation of both the frequency of filament formation and filament length [58].

The *Sc*CTPS8 isoform was also activated by GTP and, again, the requirement for GTP was not absolute [74]. Binding of GTP did not alter the enzyme’s affinity for Gln and, at a saturating concentration of Gln, GTP stimulated Gln-dependent CTP formation by 12-fold (*K*_A_ = 26 µM).

### 4.7. Drosophila melanogaster

Gln-dependent CTP synthesis catalyzed by *Dm*CTPS is activated by GTP [66]. Interestingly, the substitution of Leu 444 (Leu 435 in *Ec*CTPS) by Ala at the GTP binding site (vide infra) markedly reduced the activating effect of GTP. In addition, substitution of Ala for Phe 50 (Met 52 in *Ec*CTPS), which resides on the L2 loop (shaded yellow in Figure 2) and forms part of the NH_3_ tunnel, also markedly reduced the activating effect of GTP [66].

### 4.8. Plants

The kinetic properties of CTPSs from plants have not been extensively characterized. Interestingly, the enzyme from *Arabidopsis thaliana* (*At*CTPS) occurs as five isoforms and six putative isoforms that have been identified in rice (endospermless variant from *Oryza sativa* var. *japonica* cultivar Hwayoung) [90]. *At*CTPS1–4 are expressed throughout the plant tissues, while *At*CTPS5 is expressed only in developing embryos [91]. This enzyme also undergoes filament formation as previously demonstrated for the yeast, fruit fly, human, and *E. coli* enzymes (vide infra). GTP was shown to activate *At*CTPS3 with an increase in the concentration of GTP from 0.05 mM to a saturating concentration of 1.0 mM GTP, increasing the rate of Gln-dependent CTP formation nearly 5-fold with an apparent *K*_A_ value of ~0.25 mM.

### 4.9. Mammals

Human CTPS exists as two isoforms, hCTPS1 and hCTPS2 [109,110]. Comparison of the effects of GTP concentration on hCTPS1 and hCTPS2 activity revealed that GTP stimulated Gln-dependent CTP formation with *K*_A_ values of 1.6 and 8.0 µM, respectively, while concentrations of GTP exceeding 10 mM inhibited the activity of both isoforms [100]. Earlier studies with hCTPS isolated from homogenates of HL-60 cells revealed negative cooperativity with GTP (*n* = 0.7). However, this was a crude preparation and likely contained a mixture of isoforms [111].

Initial studies on CTPS purified 200-fold from Ehrlich ascites tumor cells (mouse) revealed that the enzyme was active in the absence of GTP and that GTP activated the enzyme ~5-fold with maximal activation occurring at 1.0 mM GTP (*K*_A_ = 50 µM) [104]. The *K*_m_ value for Gln was not significantly altered by the presence of GTP. GTP had a slight inhibitory effect on the activity with exogenous NH_3_. Follow-up studies showed that GTP accelerated the glutaminase activity of the enzyme about 2.5-fold in the absence of other substrates and about 45-fold in the presence of UTP and ATP [65]. Curiously, these authors also reported that GTP could substitute for ATP as a substrate (*K*_m_ = 2.2 mM), even after confirming by HPLC analyses that their UTP and GTP preparations were both free of any ATP impurity. GMP, GDP, and dGTP were also capable of partially activating the enzyme, with the latter compound also serving to replace ATP [105]. Finally, the non-hydrolyzable GTP analogue Gpp[NH]p was shown to activate the enzyme by stimulating the glutaminase activity only ~1.4-fold or ~1.8-fold in the absence or presence, respectively, of both UTP and App[NH]p. However, in the presence of UTP and ATP, the glutaminase activity with concomitant CTP formation was enhanced ~32-fold. These results are not unlike those obtained by Willemoës and co-workers working with *Ll*CTPS [36], indicating that the presence of UTP and ATP is required for the maximum activation of the glutaminase activity by GTP or Gpp[NH]p.

Bovine calf liver CTPS was shown to not have an absolute requirement for GTP, but in the absence of GTP, the rate was reduced ~7-fold relative to the rate observed at saturating concentrations of GTP [63,101]. The *K*_A_ value was reported to be 70 µM (*n* = 1.1), the same as the apparent *K*_A_ value accompanying GTP-dependent activation of crude preparations of rat liver CTPS (Table 2) [18]. GTP did not promote tetramerization of the enzyme [63,101,102].

Among the various species for which the kinetics of GTP-dependent activation have been examined, the *K*_A_ values range from 1.6 µM to ~250 µM (Table 2). Compared to the intracellular concentrations of GTP, which are typically 468 ± 224 µM [112], it appears that CTPSs would be operating at near maximal activation by GTP under typical physiological conditions. Since intracellular concentrations of GTP in normal human cells and human tumor cells are 232 ± 202 µM and 473 ± 214 µM [112], respectively, this appears to be especially true for hCTPS1 with the lowest *K*_A_ value reported to date [100]. However, the following observations present a possible caveat to this generalization. Using a mutant mouse T lymphoblast cell line (FURT-1A), which contained a CTPS refractory to complete inhibition by CTP, Aronow and Ullman [103] showed that exogenous manipulation of levels of GTP between 10% and 200% of that of unperturbed cells by either mycophenolic acid or guanosine revealed that the cooperativity of the enzyme with respect to GTP activation was much greater in situ (*n* = 1.59) than in vitro (*n* = 0.76). Furthermore, the concentrations of GTP required to activate the enzyme in situ were much *greater* than those in vitro.

## 5. A New Level of Regulation: Role of the GAT Domain in Forming Filamentous Structures

**Figure 4:**
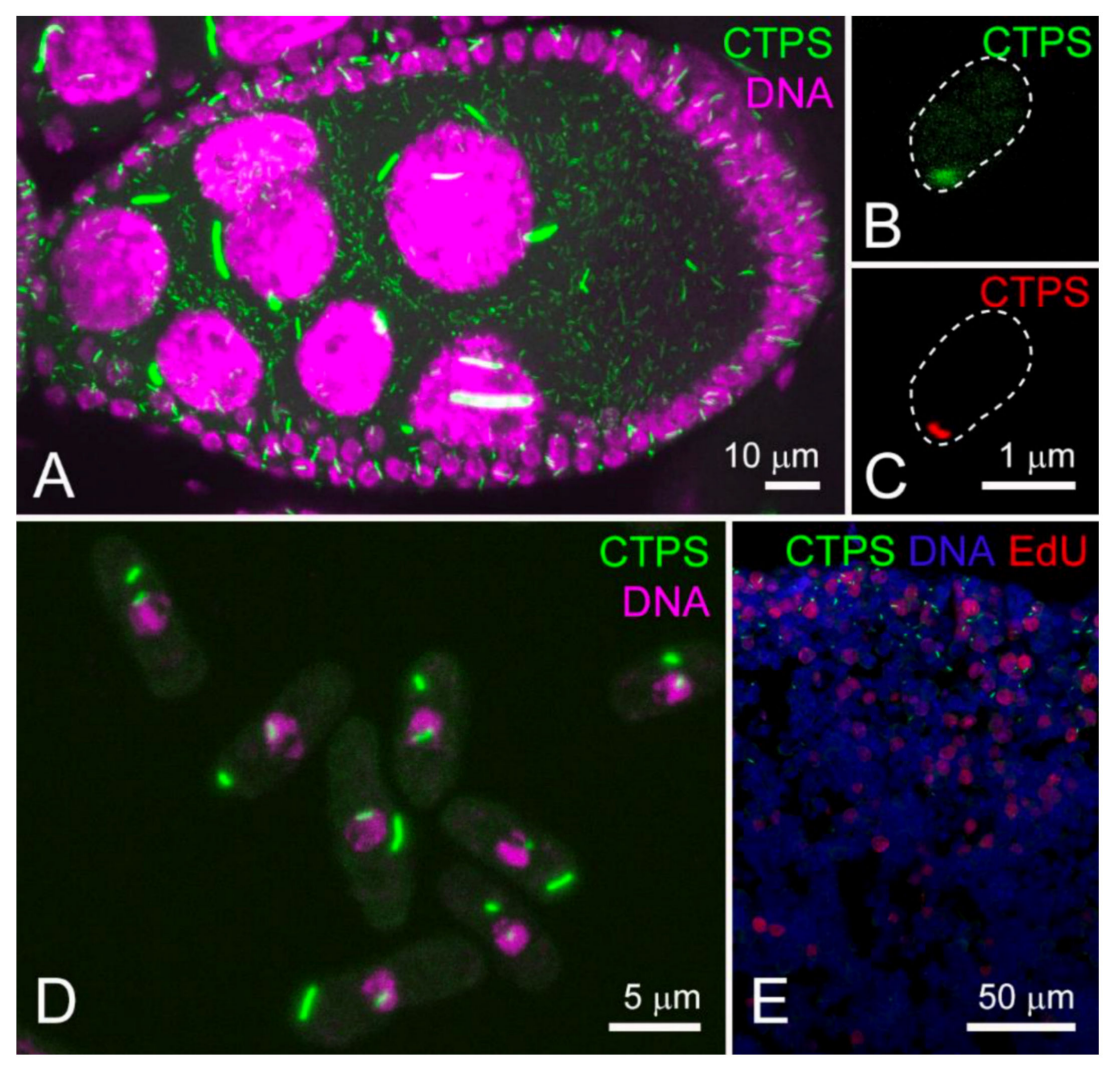
CTPS forms cytoophidia in various organisms. Intracellular filamentous structures of CTPS known as cytoophidia (green) are formed in a variety or organisms, including the *Drosophila melanogaster* egg chamber (**A**) [113], *Haloarcula hispanica* (**B**), conventional confocal image; (**C**), STED image) [117], *Schizosaccharomyces pombe* (**D**) [116], and mouse thymus (**E**) [120]. (**A**) *Drosophila* egg chamber (CTPS, green; DNA, magenta); (**B**) CTPS, green; (**C**) CTPS, red; (**D**) CTPS, green; DNA, magenta; (**E**) CTPS, green; DNA, blue; 5-ethynyl-2′-deoxyuridine (EdU, which labels proliferating cells), red. Reprinted with permission from Refs. [113], [117], and [120]. Copyright 2010, 2020, and 2021 Elsevier.

In 2010, CTPS was found to form filamentous structures termed cytoophidia (meaning “cellular snakes” in Greek) in *Drosophila melanogaster* using laser-scanning confocal microscopy [113]. Cytoophidia are widely distributed in cells from many tissues including ovaries, testes, larval brains, guts, trachea, lymph glands, and accessory glands [113,114]. Subsequent studies have shown that CTPS forms cytoophidia in bacteria [55], budding yeast [115], fission yeast [116], plants [91], archaea [117], zebrafish [118], mice [54], and humans [119], indicating that filament formation is an ancient and evolutionarily-conserved property of CTPS (Figure 4).

**Figure 5:**
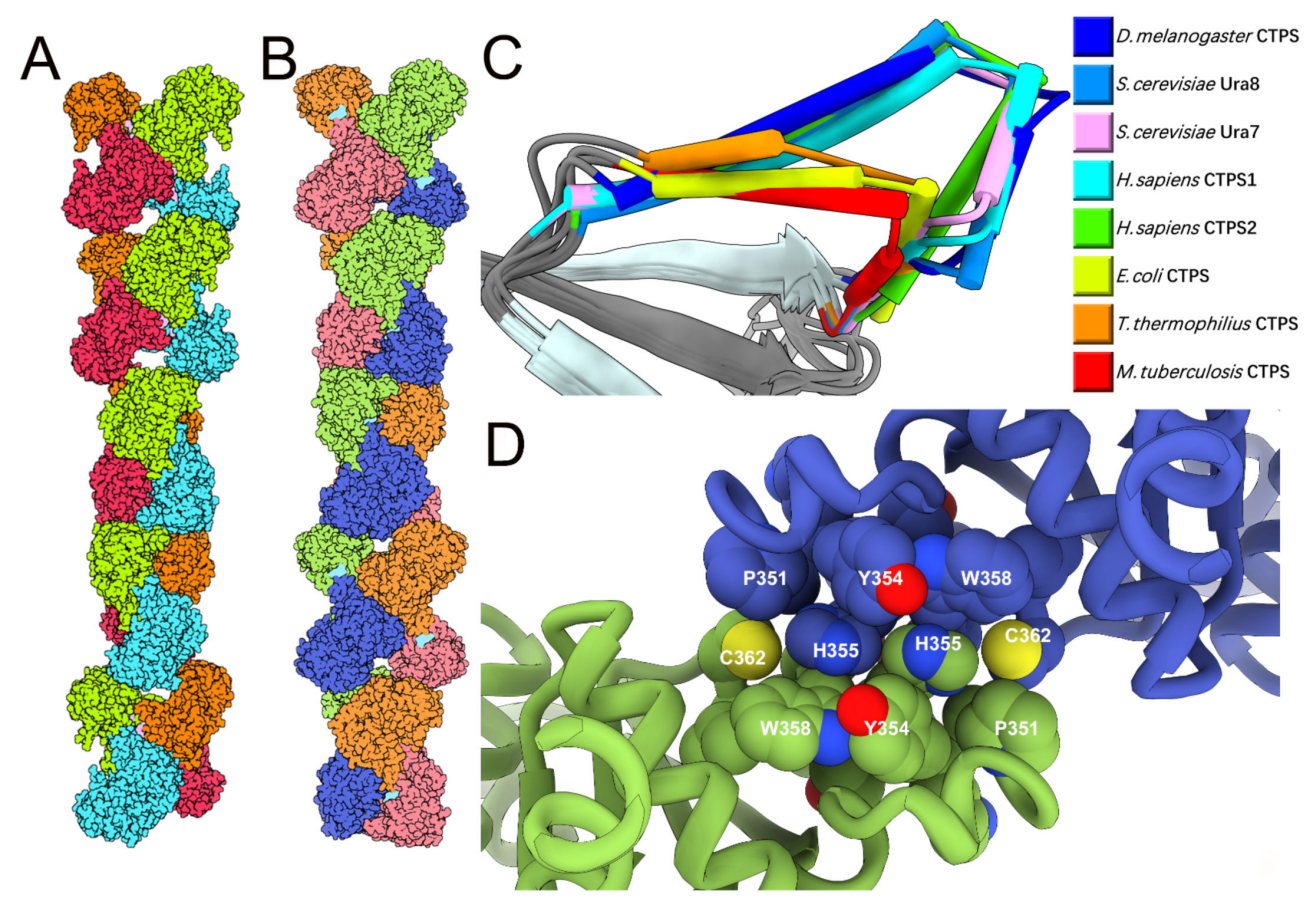
Structural features of *Dm*CTPS filaments. (**A**) Atom model display for CTPS filament assembly of tetramers for product-bound *Dm*CTPS. Protomers of a tetramer are colored differently and CTP is shaded pink. (**B**) Atom model display for CTPS filament assembly of tetramers for substrate-bound *Dm*CTPS. Protomers of a tetramer are colored differently and GTP is shaded cyan. (**C**) Comparison of the filament interaction region between β sheets 2 and 3 among different species as shown in the color key. Models are aligned by the GAT domain. β-Sheets 2 and 3 are colored in light cyan. (**D**) *Dm*CTPS filament interface. Residues at the interface are displayed and labeled. Sulfur atoms are shown as yellow spheres.

In order to explore the molecular biological basis of cytoophidia, Liu and co-workers purified *Dm*CTPS and found that the protein can form two types of filamentous structures in vitro with different conformations in the presence of substrates ATP, UTP, or product CTP (Figure 5A,B) [66]. In these two types of filaments, *Dm*CTPS is present in tetrameric units, which interact with each other through an interface in the GAT domain to form the stable filamentous structures. By comparing amino acid sequences of CTPSs, Liu and colleagues discovered that these structures are conserved among multiple species including human, fly, and yeast. At the same time, cryo-EM structures of the two CTPS isoforms in human, hCTPS1 and hCTPS2 [56,61], and the two CTPS isoforms in budding yeast, URA7 and URA8 [121], revealed that these CTPSs are assembled into filamentous structures through the same region (Figure 5C). Interestingly, there is an insertion of about 15 amino acids between β sheets 2 and 3 of the GAT domains from *Drosophila* and human CTPSs compared with the GAT domain from other class I Gln-dependent amidotransferases, which makes *Drosophila* and human GAT domains form an additional short helix and helix2 extensions. In *Dm*CTPS, the adjacent GAT domains are used to form filamentous structures through the interaction of helix2 and surrounding amino acids. Indeed, a single point mutation yielding the H355A *Dm*CTPS variant is sufficient to disrupt this interaction and affect the formation of filamentous structure (Figure 5D) [66].

This role of the GAT domain in filament forming ability obtained through amino acid insertion undoubtedly provides a new level for the regulation of the enzyme’s catalytic activity [56,61,66,121]; however, the role of filament formation on the GAT domain itself and its regulation of catalytic activity in the GAT domain remain unclear. In budding yeast, the GAT domains of URA7 and URA8 are bound with different strengths and interact via different amino acids at this insertion [121]. Therefore, regulation of the activity of the GAT domain through the insertion in this region may vary between species.

## 6. CTPS with Bound GTP: Confirmation of the Location of the GTP-Binding Site and Relationship to Other Cryo-EM Structures

**Figure 6:**
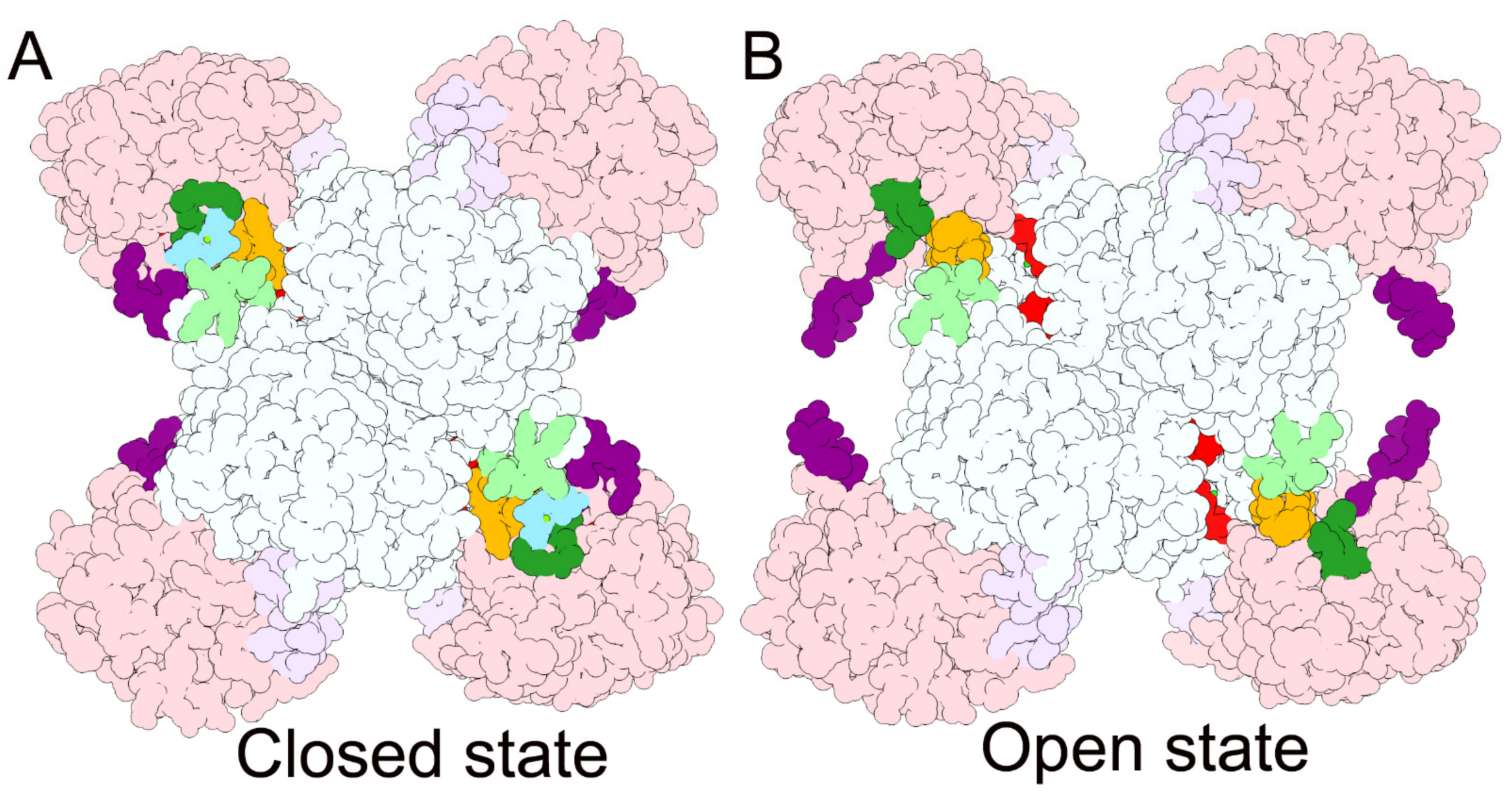
The location of GTP-binding site. Atom model display for *Dm*CTPS in a closed state with GTP bound (**A**) and in an open state without GTP bound (**B**) [66]. The GAT domain, interdomain linker, and the synthase domain are colored pink, light purple, and white, respectively. Regions interacting with GTP are colored as orange for the gate, dark green for F373 and the lid L11, light green for loop 105–110 (L4), purple for the “wing” (L13). GTP and the 4-phospho-UTP intermediate are colored blue and red in panel A, respectively. CTP is colored red in panel B.

**Figure 7:**
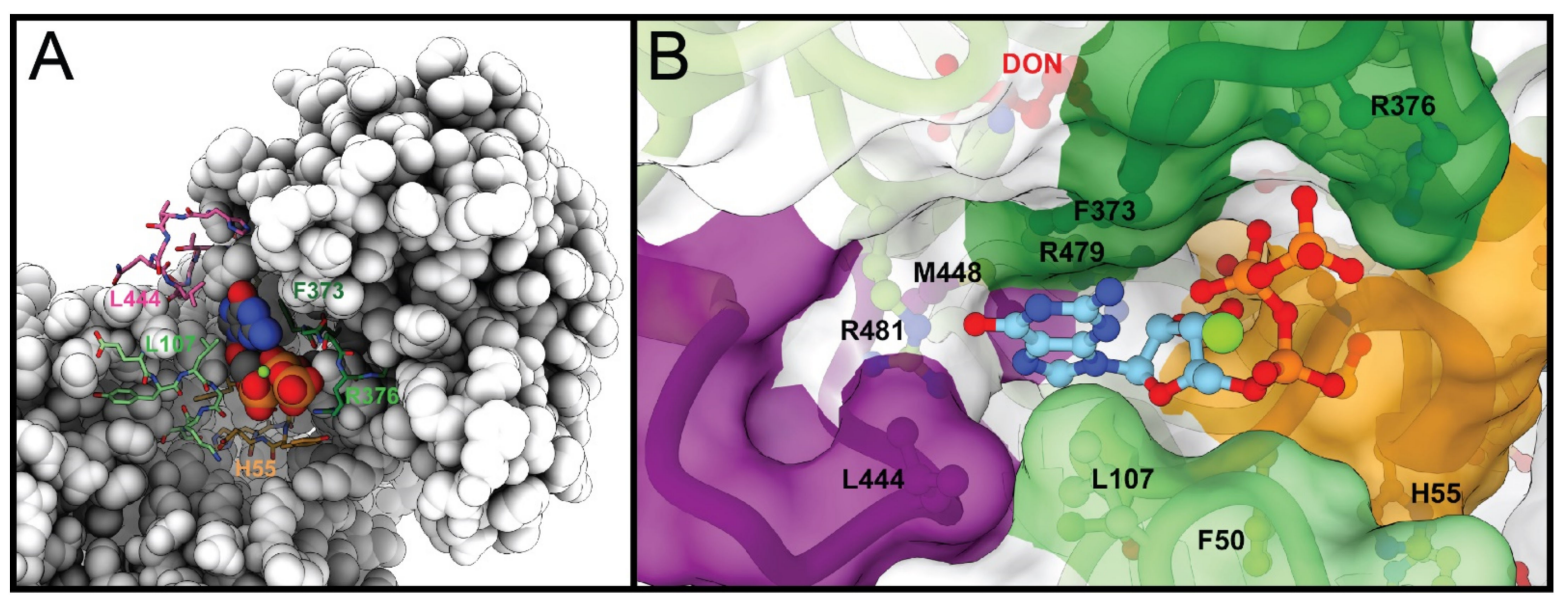
Atomic model of interaction between GTP and *Dm*CTPS. (**A**) *Dm*CTPS and GTP·Mg^2+^ are shown in space-filling representation from the cryo-EM structure solved by Liu and co-workers [66]. Shown in stick representation are residues implicated in GTP-dependent activation of CTPS, which are highlighted in light green (L4), dark green (L11), and purple (L13 or “wing”) (*cf*. coloring in Figure 2), and orange (gate region). (**B**) GTP-binding pocket of *Dm*CTPS is depicted by a transparent surface. GTP and DON are shown in ball and stick representation, as are residues that interact with GTP or play a role in GTP-dependent conformational effects: F50, H55, L107, F373, R376, L444, M448, R429, and R481 (colored as in Figure 2). The Mg^2+^ ion is shown as a light green sphere in both panels.

Despite the various kinetics and site-directed mutagenesis experiments conducted to delineate the structural and functional aspects of the GTP-dependent regulation of CTPS activity, specific structural information on the GTP binding site and the conformational changes that accompanied GTP binding remained elusive. In 2021, the first structure of CTPS with bound GTP was captured using cryo-EM (Figure 6) [66]. This structure of *Dm*CTPS was covalently modified at the GAT domain by the Gln analogue DON and revealed a tightly closed state with the CTPS tetramers forming a filamentous structure through the amino acid insertion mentioned above. As predicted by Baldwin and co-workers [32], GTP is bound in a cleft between the GAT domain and the synthase domain (Figure 3). The GTP molecule is tightly surrounded by the gate (which forms a part of the NH_3_ tunnel), lid L11, Phe 373 (which encapsulates the catalytic triad of the GAT domain), and the L4 loop 105–110 of an adjacent protomer (Figure 7). Compared with other CTPS structures in the tightly closed state, the DON-modified tetramer with bound GTP exhibits an even tighter conformation, which may be related to the formation and tight connection of the NH_3_ tunnel. Intriguingly, Liu and co-workers also observed the presence of 4-phosphorylated UTP intermediate in their cryo-EM structure of *Dm*CTPS [66]. This observation suggests that the first step of the catalytic reaction (i.e., ATP-dependent phosphorylation of UTP) has been completed in the synthase domain, and the intermediate is waiting for NH_3_ to be transferred from the GAT domain to complete the final amination reaction.

GTP also plays an important role in the formation of the NH_3_ tunnel. Upon simulating the removal of GTP from their structure, Liu and co-workers found that there is a hole in the NH_3_ tunnel adjacent to where GTP binds, as originally suggested by Baldwin and co-workers [32]. This observation indicates that GTP can indeed prevent leakage of NH_3_ from the ammonia tunnel and appears to account for why GTP inhibits the reaction with exogenous NH_3_ when CTPS is covalently modified by DON, as shown in previous studies [36,64,86]. Interestingly, the NH_3_ tunnel formed in GTP-bound *Dm*CTPS is narrower than that in CTPS bound with the products or some other substrates.

Compared with carbamoyl-phosphate synthetase 2, which is also a class I Gln-dependent amidotransferase, there is an insertion of about eight amino acids between β sheets 5 and 6 in the GAT domain of CTPS, which has been referred to as the “wing” (residues 440–448 in *Dm*CTPS and 431–439 in *Ec*CTPS; L13 purple region in Figure 2) [66]. Many of the previous crystal and cryo-EM structures of CTPSs have shown that this wing is very flexible, making it very challenging to determine its precise structure and thereby limiting our understanding of its role in GTP-dependent activation. Utilizing DON-modified *Dm*CTPS with bound GTP, Liu and co-workers were able to obtain a clear structure of the wing of *Dm*CTPS using cryo-EM [66]. The wing directly contacts GTP, suggesting that GTP binding evokes a conformational change that stabilizes the wing region. Similar to the R429A *Ec*CTPS variant [81] and the T431V and R433A *Ll*CTPS variants [85], the L444A substitution obviated the regulatory effect of GTP on *Dm*CTPS activity [66]. This further shows that the integrity of the wing is necessary for the GTP-dependent regulation of CTPS activity and furnishes a structural explanation for the results obtained from previous kinetics studies (Section 2.3.1).

## 7. A Model for GTP-Dependent Regulation of CTPS

With the report of the GTP-bound structure of *Dm*CTPS [66], combined with the results from the site-directed mutagenesis and kinetics studies described in Section 2 and Section 3, a more comprehensive model for the role of GTP in regulating CTPS catalysis emerges. While the model described here incorporates features observed for CTPSs from different organisms, subtle differences likely exist between various CTPSs. As Liu and co-workers noted, the initial binding of UTP and ATP effects the open-to-closed state transition that alters the conformation of the GTP-binding cleft to facilitate GTP binding [66]. Concomitantly, ATP and UTP binding cause a rotation of the GAT and synthase domains to align their active sites for formation of the NH_3_ tunnel [56]. In *Ll*CTPS, Willemoës and co-workers proposed that GTP binding not only closes a lid over the active site to effect proficient hydrolysis of Gln, but there is also concomitant formation of the 4-phospho-UTP intermediate, which causes allosteric activation of the glutaminase activity [36]. This reciprocal activating effect of GTP and the intermediate is consistent with the observed presence of the 4-phospho-UTP intermediate in the GTP-bound structure of DON-modified *Dm*CTPS [66].

Within the GAT domain, a series of interactions contribute to altering the conformation of the GAT domain. Gln binding orders Phe 373 (*Dm*CTPS numbering, 353 in *Ec*CTPS) of the L11 lid to afford an additional stabilizing interaction with bound GTP. In addition to interacting with GTP, Leu 107 (109 in *Ec*CTPS), from the L4 region of an adjacent protomer, interacts with Leu 444 (435 in *Ec*CTPS) located in the unique wing structure (L13) in the GAT domain to propagate the conformational change accompanying GTP binding. Arg 376 (Arg 359 in *Ll*CTPS) interacts with GTP and appears to act as a lever to alter the conformation of the lid L11 to favor Gln hydrolysis [84]. The interaction between Phe 50 and GTP appears to play a role in decreasing the flexibility of the L2 region, thereby ensuring the integrity of the NH_3_ tunnel while GTP is bound. The hole from which the nascent NH_3_ could be lost to bulk solvent is effectively “capped” by the bound GTP, ensuring the fidelity of the transfer of NH_3_ to the synthase domain.

Thus, GTP not only promotes the hydrolysis of Gln through its multiple interactions with the GAT domain of CTPS, but also participates in the assembly and maintenance of the NH_3_ tunnel, and thereby coordinates the reactions catalyzed by the GAT and synthase domains of the enzyme. Although direct experimental evidence on the specific mechanism for GTP-dependent regulation of catalysis at the GAT domain of CTPS is still lacking, we posit that the GAT domain in CTPS forms a unique regulatory system around GTP binding that is both efficient and sensitive.

## Figures and Tables

**Table 1:** CTPS variants implicating conformational changes accompanying allosteric activation by GTP.

Variant	Organism	Observations (Changes in Kinetic Parameters Are Relative to Wild-Type Enzyme)			
(*E. coli* Residue), Structural Element ^a^		NH_3_-Dependent CTP Formation	GTP-Dependent Activation of Gln-Dependent CTP Formation	Glutaminase Activity (in the Presence of GTP)	Reference
R105A, L4	*E. coli*	no change in *k*_cat_ no change in *K*_m_	↓ *k*_cat_ 4.0-fold ↑ *K*_m_ 1.2-fold	↓ *k*_cat_ 2.5-fold ↑ *K*_A_ 3.1-fold	[82]
D107A, L4	*E. coli*	no change in *k*_cat_ ↓ *K*_m_ 1.2-fold	↓ *k*_cat_ 1.7-fold ↓ *K*_m_ 1.3-fold	↓ *k*_cat_ 1.2-fold ↑ *K*_A_ 1.6-fold	[82]
L109A, L4	*E. coli*	↓ *k*_cat_ 1.2-fold ↓ *K*_m_ 1.3-fold	↓ *k*_cat_ 3.7-fold ↑ *K*_m_ 1.2-fold	no change in *k*_cat_ ↑ *K*_A_ 3.8-fold	[82]
L109A, L4 (NH_2_OH as substrate)	*E. coli*	no change in *k*_cat_ no change in *K*_m_	↓ *k*_cat_ 7.2-fold ^b^ ↑ *K*_m_ 1.5-fold ^b^	↓ *k*_cat_ 3.0-fold ^b^ ↓ *K*_m_ 1.2-fold ^b^	[83]
L109F, L4	*E. coli*	↑ *k*_cat_ 1.2-fold no change in *K*_m_	↓ *k*_cat_ 5.9-fold no change in *K*_m_	↓ *k*_cat_ 5.8-fold ↑ *K*_A_ 4.9-fold	[82]
R429A, L13	*E. coli*	no change in *k*_cat_ no change in *K*_m_	↓ *k*_cat_ 15-fold ↑ *K*_m_ 1.7-fold	↓ *k*_act_ 6.0-fold ↑ *K*_A_ 12-fold	[81]
G352P, L11	*E. coli*	↓ specific activity 1.3-fold	no activity observed	–	[31]
R359M (R356, L11)	*L. lactis*	no change in *k*_cat_ no change in *K*_m_	↓ *k*_act_ 12-fold ↑ *K*_A_ 4.6-fold	↓ *k*_cat_ 1.3-fold ↑ *K*_m_ 1.2-fold	[84]
R359P (R356, L11)	*L. lactis*	↓ *k*_cat_ 1.2-fold ↑ *K*_m_ 1.7-fold	↓ *k*_cat_~43-fold ^c^ ↑ *K*_A_~11-fold ^c^	↓ *k*_cat_ 4.3-fold no change in *K*_m_	[84]
G360A (G357, L11)	*L. lactis*	↑ *k*_cat_ 1.2-fold no change in *K*_m_	↑ *k*_act_ 1.3-fold ↓ *K*_A_ 1.2-fold	↑ *k*_cat_ 1.8-fold ↑ *K*_m_ 1.2-fold ↑ *k*_cat_ 1.6-fold ^e^ no change in *K*_A_^e^	[84]
G360P (G357, L11)	*L. lactis*	↓ *k*_cat_ 3.5-fold ↑ *K*_m_ 1.7-fold	↓ *k*_cat_~24-fold ^c,d^ ↑ *K*_A_ 142-fold ^c^	–	[84]
E362Q (E359, L11-α13)	*L. lactis*	↑ *k*_cat_ 1.6-fold ↑ *K*_m_ 1.4-fold	↑ *k*_act_ 1.2-fold no change in *K*_A_	↓ *k*_cat_ 4.3-fold no change in *K*_m_	[84]
T431V (T438, L13-β18)	*L. lactis*	↑ *k*_cat_ 1.2-fold ↑ *K*_m_ 1.7-fold	↓ *k*_cat_ 74-fold ↓ *k*_act_ 104-fold no change in *K*_A_	↓ *k*_cat_ 2.8-fold no change in *K*_m_	[85]
R433M (R440, β18)	*L. lactis*	↓ *k*_cat_ 2-fold no change in *K*_m_	↓ *k*_cat_ 8.4-fold ↓ *k*_act_ 12.7-fold ↓ *K*_A_ 17-fold	↓ *k*_cat_ 1.6-fold ↓ *K*_m_ 1.2-fold	[85]
F50A (M52, L2)	*D. melanogaster*	NR	qualitatively markedly reduced with 0.2 and 2 mM GTP	NR ^f^	[66]
L444A (L435, L13)	*D. melanogaster*	NR	qualitatively markedly reduced with 0.2 and 2 mM GTP	NR	[66]
R381M (R356, L11)	*S. cerevisiae*	NR	% filaments, average filament length, and median filament length: 86%, 1.37 µm, and 1.41 µm ^g^	NR	[58]
R381P (R356, L11)	*S. cerevisiae*	NR	% filaments, average filament length, and median filament length: 82%, 1.20 µm, and 1.022 µm ^g^	NR	[58]
G382A (G357, L11)	*S. cerevisiae*	NR	% filaments, average filament length, and median filament length: 54%, 0.83 µm, and 0.79 µm ^g^	NR	[58]

^a^ As defined for *Ec*CTPS in [32]; ^b^ Gln-OH as the substrate [83]; ^c^ estimates only since determination was limited for technical reasons [84]; ^d^ value is an apparent value since the [Gln] may not be saturating under the experimental conditions [84]; ^e^ in the presence of ATPγS (1 mM) and UTP (1 mM) [84]; ^f^ NR = not reported; ^g^ compared with wild-type enzyme where the % filaments, average filament length, and median filament length were 69%, 1.05 µm, and 1.06 µm, respectively [58]. Arrows indicate the direction of the change.

**Table 2:** Effect of GTP and analogues on CTPS activity.

Organism, Isoform(s)	*K*_A_, µM	Fold Increase ^a^	Other Activators and Inhibitors	Additional Observations	References
**Prokaryotes**					
*Escherichia coli*, *Ec*CTPS	81 ^c^ 32 ^d^	↑7.1 × ^b^ ↑15 × ^c^ ↑51 × ^d^	6-thio-GTP (*k*_act_ = 8.5 s^−1^, *K*_A_ = 35 µM) ^c^ GtetraP (*k*_act_ = 4.0 s^−1^, *K*_A_ = 190 µM) ^c^ ITP (*k*_act_ = 5.2 s^−1^, *K*_A_ = 2900 µM) ^c^ *O*^6^-Me-GTP (*k*_act_ = 2.8 s^−1^, *K*_A_ = 130 µM) ^c^ 2′-dGTP (*k*_act_ = 1.5 s^−1^, *K*_A_ = 210 µM) ^c^	[S]_0.5_ (Gln) decreased 6.3-fold ^b^ For GTP: ^c^ *k*_o_ = 0.73 s^−1^, *k*_act_ = 10.6 s^−1^, *K*_i_ = 280 µM, *n* = 4.0 For GTP: ^d^ *k*_o_ = 0.14 s^−1^, *k*_act_ = 7.1 s^−1^	[64,72,81]
*Lactococcus lacti*, *Ll*CTPS	31 ^e^ 136 ^f^ 2.7 ^g^ 220 ^f^ 2430 ^h^ 1620 ^i^	↑25 × ^e^ ↑50 × ^f^ ↑7 × ^g^ ↑49 × ^f^ ↑15 × ^h^ ↑14 × ^i^	Inhibition of glutaminase rxn. with 0.1 mM UTP and ATPγS at [GTP] > 2 mM	(*k*_cat,1_ = 0.028 s^−1^, *k*_cat,2_ = 0.195) ^d^ (*k*_cat,1_ = 0.130 s^−1^, *k*_cat,2_ = 6.4) ^c^ (*k*_cat,1_ = 0.078 s^−1^, *k*_cat,2_ = 1.20) ^e^ (*k*_cat,1_ = 0.76 s^−1^, *k*_cat,2_ = 1.054) ^f^	[36,73]
*Thermus thermophilus*, *Tt*CTPS	–	–	–	*n* = 2.1	[45]
**eukaryotes**					
*Trypanosomal brucei*, *Tb*CTPS	70	↑5.4 ×	Inhibitors: GTP (IC_50_ = 460 µM), guanosine (IC_50_ = 380 µM), caffeine (IC_50_ = 480 µM), uric acid (IC_50_ = 100 µM)	*k*_o_ = 0.16 s^−1^ *k*_act_ = 0.87 s^−1^ *K*_A_ = 57 µM *K*_i_ = 272 µM *n* = 4.2	[9,14]
*Arabidopsis thaliana*, *At*CTPS	~250	↑5 ×	–	–	[90,91]
*Chlamydia trachomatis*, *Ct*CTPS	7	–	–	–	[92]
*Plasmodium falciparum*, *Pf*CTPS	–	–	–	noted increase in rate, but no kinetic data	[10,93]
*Giardia intestinalis*, *Gi*CTPS	60 (400) ^j^	–	Activators: GTP > GDP (*K*_A_ = 100 µM) > dGTP (*K*_A_ = 230 µM)	–	[12,94,95]
*Saccharomyces cerevisiae*, *Sc*CTPS7	15.3–15.7	↑4 ×	–	*K*_A_ = 12.0 µM and 12.2 µM when phosphorylated by PKA and PKC	[58,96,97,98,99]
*Sc*CTPS8	26	↑12 ×	–	–	[74]
**mammalian**					
*Homo sapiens*, hCTPS1	1.6	↑4 ×	Inhibited by [GTP] > 10 mM	*–*	[100]
hCTPS2	8.0	↑12 ×	Inhibited by [GTP] > 10 mM	–	[100]
*Bos taurus*, calf liver CTPS	70	↑7 ×	–	*n* = 1	[63,101,102]
*Rattus norvegicus*, rat liver CTPS	70	–	–	–	[18]
*Mus musculus*, *Mm*CTPS from FURT-A cells	–	–	Intracellular GTP levels varied between 10% and 200% of that of unperturbed cells showed that the cooperativity of *Mm*CTPS for GTP activation differed: *n* = 1.59 (in situ) vs. *n* = 0.76 (in vitro)	[GTP] required to activate *Mm*CTPS in situ > than that in vitro	[103]
*Mus musculus*, Ehrlich ascites tumor cells	50	↑5 ×	Activators: GMP, GDP, dGTP, Gpp[NH]p, GTP acts as a substrate!	↑1.4–2.5 × glutaminase activity in the absence of ATP & UTP, ↑32–45 × in the presence	[65,104,105]

^a^ enhancement of Gln-dependent CTP formation by GTP; ^b^ from [64]; ^c^ from [72]; ^d^ from [81]; ^e^ [ATP] = [UTP] = 0.25 mM; ^f^ [ATP] = [UTP] = 1.0 mM; ^g^ [ATP] = [UTP] = 0.1 mM; ^h^ [ATPγS] = [UTP] = 0.1 mM; ^i^ [ATPγS] = [UTP] = 1.0 mM; ^j^ limited and noisy data in constructing hyperbolic plot [95]. Arrows denote changes in activity.

## Data Availability

Not applicable.

## References

[1] Bakovic M., Fullerton M.D., Michel V. (2007). Metabolic and molecular aspects of ethanolamine phospholipid biosynthesis: The role of CTP: Phosphoethanolamine cytidylyltransferase (Pcyt2). Biochem. Cell Biol..

[2] Chang Y.F., Carman G.M. (2008). CTP synthetase and its role in phospholipid synthesis in the yeast *Saccharomyces cerevisiae*. Prog. Lipid Res..

[3] Ostrander D.B., O’Brien D.J., Gorman J.A., Carman G.M. (1998). Effect of CTP synthetase regulation by CTP on phospholipid synthesis in *Saccharomyces cerevisiae*. J. Biol. Chem..

[4] Hatse S., De Clercq E., Balzarini J. (1999). Role of antimetabolites of purine and pyrimidine nucleotide metabolism in tumor cell differentiation. Biochem. Pharmacol..

[5] Shridas P., Waechter C.J. (2006). Human dolichol kinase, a polytopic endoplasmic reticulum membrane protein with a cytoplasmically oriented CTP-binding site. J. Biol. Chem..

[6] Rivera-Serrano E.E., Gizzi A.S., Arnold J.J., Grove T.L., Almo S.C., Cameron C.E. (2020). Viperin reveals its true function. Ann. Rev. Virol..

[7] De Clercq E. (1993). Antiviral agents: Characteristic activity spectrum depending on the molecular target with which they interact. Adv. Virus. Res..

[8] De Souza J.O., Dawson A., Hunter W.N. (2017). An improved model of the *Trypanosoma brucei* CTP synthetase glutaminase domain:acivicin complex. Chem. Med. Chem..

[9] Fijolek A., Hofer A., Thelander L. (2007). Expression, purification, characterization, and in vivo targeting of trypanosome CTP synthetase for treatment of African sleeping sickness. J. Biol. Chem..

[10] Hendriks E.F., O’Sullivan W.J., Stewart T.S. (1998). Molecular cloning and characterization of the *Plasmodium falciparum* cytidine triphosphate synthetase gene. Biochim. Biophys. Acta.

[11] Hofer A., Steverding D., Chabes A., Brun R., Thelander L. (2001). *Trypanosoma brucei* CTP synthetase: A target for the treatment of African sleeping sickness. Proc. Natl. Acad. Sci. USA.

[12] Lim R.L., O’Sullivan W.J., Stewart T.S. (1996). Isolation, characterization and expression of the gene encoding cytidine triphosphate synthetase from *Giardia intestinalis*. Mol. Biochem. Parasitol..

[13] Narvaez-Ortiz H.Y., Lopez A.J., Gupta N., Zimmermann B.H. (2018). A CTP synthase undergoing stage-specific spatial expression is essential for the survival of the intracellular parasite *Toxoplasma gondii*. Front. Cell. Infect. Microbiol..

[14] Steeves C.H., Bearne S.L. (2011). Activation and inhibition of CTP synthase from *Trypanosoma brucei*, the causative agent of African sleeping sickness. Bioorg. Med. Chem. Lett..

[15] Mori G., Chiarelli L.R., Esposito M., Makarov V., Bellinzoni M., Hartkoorn R.C., Degiacomi G., Boldrin F., Ekins S., de Jesus Lopes Ribeiro A.L. (2015). Thiophenecarboxamide derivatives activated by EthA kill *Mycobacterium tuberculosis* by inhibiting the CTP synthetase PyrG. Chem. Biol..

[16] Esposito M., Szadocka S., Degiacomi G., Orena B.S., Mori G., Piano V., Boldrin F., Zemanová J., Huszár S., Barros D. (2017). A phenotypic based target screening approach delivers new antitubercular CTP synthetase inhibitors. ACS Infect. Dis..

[17] Chiarelli L.R., Mori G., Orena B.S., Esposito M., Lane T., de Jesus Lopes Ribeiro A.L., Degiacomi G., Zemanová J., Szádocka S., Huszár S. (2018). A multitarget approach to drug discovery inhibiting *Mycobacterium tuberculosis* PyrG and PanK. Sci. Rep..

[18] Williams J.C., Kizaki H., Weber G., Morris H.P. (1978). Increased CTP synthetase activity in cancer cells. Nature.

[19] Kizaki H., Williams J.C., Morris H.P., Weber G. (1980). Increased cytidine 5′-triphosphate synthetase activity in rat and human tumors. Cancer Res..

[20] Kang G.J., Cooney D.A., Moyer J.D., Kelley J.A., Kim H.Y., Marquez V.E., Johns D.G. (1989). Cyclopentenylcytosine triphosphate. Formation and inhibition of CTP synthetase. J. Biol. Chem..

[21] Van den Berg A.A., van Lenthe H., Busch S., de Korte D., Roos D., van Kuilenburg A.B., van Gennip A.H. (1993). Evidence for transformation-related increase in CTP synthetase activity in situ in human lymphoblastic leukemia. Eur. J. Biochem..

[22] Van den Berg A.A., van Lenthe H., Busch S., de Korte D., van Kuilenburg A.B., van Gennip A.H. (1994). The roles of uridine-cytidine kinase and CTP synthetase in the synthesis of CTP in malignant human T-lymphocytic cells. Leukemia.

[23] Viola J.J., Agbaria R., Walbridge S., Oshiro E.M., Johns D.G., Kelley J.A., Oldfield E.H., Ram Z. (1995). In situ cyclopentenyl cytosine infusion for the treatment of experimental brain tumors. Cancer Res..

[24] Agbaria R., Kelley J.A., Jackman J., Viola J., Ram Z., Oldfield E., Johns D.G. (1997). Antiproliferative effects of cyclopentenyl cytosine (NSC 375575) in human glioblastoma cells. Oncol. Res..

[25] Verschuur A.C., Van Gennip A.H., Leen R., Voute P.A., Brinkman J., Van Kuilenburg A.B. (2002). Cyclopentenyl cytosine increases the phosphorylation and incorporation into DNA of 1-β-D-arabinofuranosyl cytosine in a human T-lymphoblastic cell line. Int. J. Cancer.

[26] Lin Y., Zhang J., Li Y., Guo W., Chen L., Chen M., Chen X., Zhang W., Jin X., Jiang M. (2022). CTPS1 promotes malignant progression of triple-negative breast cancer with transcriptional activation by YBX1. J. Transl. Med..

[27] Sun Z., Zhang Z., Wang Q.-Q., Liu J.-L. (2022). Combined inactivation of CTPS1 and ATR is synthetically lethal to MYC-overexpressing cancer cells. Cancer Res..

[28] Martin E., Palmic N., Sanquer S., Lenoir C., Hauck F., Mongellaz C., Fabrega S., Nitschké P., Esposti M.D., Schwartzentruber J. (2014). CTP synthase 1 deficiency in humans reveals its central role in lymphocyte proliferation. Nature.

[29] Lynch E.M., DiMattia M.A., Albanese S., van Zundert G.C.P., Hansen J.M., Quispe J.D., Kennedy M.A., Verras A., Borrelli K., Toms A.V. (2021). Structural basis for isoform-specific inhibition of human CTPS1. Proc. Natl. Acad. Sci. USA.

[30] Zalkin H. (1993). The amidotransferases. Adv. Enzymol. Relat. Areas Mol. Biol..

[31] Weng M.L., Zalkin H. (1987). Structural role for a conserved region in the CTP synthetase glutamine amide transfer domain. J. Bacteriol..

[32] Endrizzi J.A., Kim H., Anderson P.M., Baldwin E.P. (2004). Crystal structure of *Escherichia coli* cytidine triphosphate synthetase, a nucleotide-regulated glutamine amidotransferase/ATP-dependent amidoligase fusion protein and homologue of anticancer and antiparasitic drug targets. Biochemistry.

[33] Levitzki A., Koshland D.E. (1971). Cytidine triphosphate synthetase. Covalent intermediates and mechanisms of action. Biochemistry.

[34] Lewis D.A., Villafranca J.J. (1989). Investigation of the mechanism of CTP synthetase using rapid quench and isotope partitioning methods. Biochemistry.

[35] Von der Saal W., Anderson P.M., Villafranca J.J. (1985). Mechanistic investigations of *Escherichia coli* cytidine-5′-triphosphate synthetase. Detection of an intermediate by positional isotope exchange experiments. J. Biol. Chem..

[36] Willemoës M., Sigurskjold B.W. (2002). Steady-state kinetics of the glutaminase reaction of CTP synthase from *Lactococcus lactis*. Eur. J. Biochem..

[37] Long C.W., Pardee A.B. (1967). Cytidine triphosphate synthetase of *Escherichia coli* B. I. Purification and kinetics. J. Biol. Chem..

[38] Chakraborty K.P., Hurlbert R.B. (1961). Role of glutamine in the biosynthesis of cytidine nucleotides in *Escherichia coli*. Biochim. Biophys. Acta.

[39] Levitzki A., Koshland D.E. (1969). Negative cooperativity in regulatory enzymes. Proc. Natl. Acad. Sci. USA.

[40] Levitzki A., Koshland D.E. (1972). Ligand-induced dimer-to-tetramer transformation in cytosine triphosphate synthetase. Biochemistry.

[41] Anderson P.M. (1983). CTP synthetase from *Escherichia coli*: An improved purification procedure and characterization of hysteretic and enzyme concentration effects on kinetic properties. Biochemistry.

[42] Thomas P.E., Lamb B.J., Chu E.H. (1988). Purification of cytidine-triphosphate synthetase from rat liver, and demonstration of monomer, dimer and tetramer. Biochim. Biophys. Acta.

[43] Pappas A., Yang W.L., Park T.S., Carman G.M. (1998). Nucleotide-dependent tetramerization of CTP synthetase from Saccharomyces cerevisiae. J. Biol. Chem..

[44] Endrizzi J.A., Kim H., Anderson P.M., Baldwin E.P. (2005). Mechanisms of product feedback regulation and drug resistance in cytidine triphosphate synthetases from the structure of a CTP-inhibited complex. Biochemistry.

[45] Goto M., Omi R., Nakagawa N., Miyahara I., Hirotsu K. (2004). Crystal structures of CTP synthetase reveal ATP, UTP, and glutamine binding sites. Structure.

[46] Choi M.G., Park T.S., Carman G.M. (2003). Phosphorylation of *Saccharomyces cerevisiae* CTP synthetase at Ser424 by protein kinases A and C regulates phosphatidylcholine synthesis by the CDP-choline pathway. J. Biol. Chem..

[47] Park T.S., O’Brien D.J., Carman G.M. (2003). Phosphorylation of CTP synthetase on Ser36, Ser330, Ser354, and Ser454 regulates the levels of CTP and phosphatidylcholine synthesis in *Saccharomyces cerevisiae*. J. Biol. Chem..

[48] Chang Y.F., Martin S.S., Baldwin E.P., Carman G.M. (2007). Phosphorylation of human CTP synthetase 1 by protein kinase C: Identification of Ser(462) and Thr(455) as major sites of phosphorylation. J. Biol. Chem..

[49] Choi M.G., Carman G.M. (2007). Phosphorylation of human CTP synthetase 1 by protein kinase A: Identification of Thr455 as a major site of phosphorylation. J. Biol. Chem..

[50] Higgins M.J., Graves P.R., Graves L.M. (2007). Regulation of human cytidine triphosphate synthetase 1 by glycogen synthase kinase 3. J. Biol. Chem..

[51] Jia F., Chi C., Han M. (2020). Regulation of nucleotide metabolism and germline proliferation in response to nucleotide imbalance and genotoxic stresses by EndoU nuclease. Cell Rep..

[52] Aughey G.N., Grice S.J., Liu J.L. (2016). The interplay between Myc and CTP synthase in *Drosophila*. PLoS Genet..

[53] Barry R.M., Bitbol A.F., Lorestani A., Charles E.J., Habrian C.H., Hansen J.M., Li H.J., Baldwin E.P., Wingreen N.S., Kollman J.M. (2014). Large-scale filament formation inhibits the activity of CTP synthetase. eLife.

[54] Gou K.M., Chang C.C., Shen Q.J., Sung L.Y., Liu J.L. (2014). CTP synthase forms cytoophidia in the cytoplasm and nucleus. Exp. Cell Res..

[55] Ingerson-Mahar M., Briegel A., Werner J.N., Jensen G.J., Gitai Z. (2010). The metabolic enzyme CTP synthase forms cytoskeletal filaments. Nat. Cell Biol..

[56] Lynch E.M., Hicks D.R., Shepherd M., Endrizzi J.A., Maker A., Hansen J.M., Barry R.M., Gitai Z., Baldwin E.P., Kollman J.M. (2017). Human CTP synthase filament structure reveals the active enzyme conformation. Nat. Struct. Biol..

[57] McCluskey G.D., Bearne S.L. (2018). Biophysical analysis of bacterial CTP synthase filaments formed in the presence of the chemotherapeutic metabolite gemcitabine-5′-triphosphate. J. Mol. Biol..

[58] Noree C., Monfort E., Shiau A.K., Wilhelm J.E. (2014). Common regulatory control of CTP synthase enzyme activity and filament formation. Mol. Biol. Cell.

[59] Strochlic T.I., Stavrides K.P., Thomas S.V., Nicolas E., O’Reilly A.M., Peterson J.R. (2014). Ack kinase regulates CTP synthase filaments during *Drosophila* oogenesis. EMBO Rep..

[60] Wang P.Y., Lin W.C., Tsai Y.C., Cheng M.L., Lin Y.H., Tseng S.H., Chakraborty A., Pai L.M. (2015). Regulation of CTP synthase filament formation during DNA endoreplication in drosophila. Genetics.

[61] Lynch E.M., Kollman J.M. (2020). Coupled structural transitions enable highly cooperative regulation of human CTPS2 filaments. Nat. Struct. Mol. Biol..

[62] Chakraborty A., Lin W.C., Lin Y.T., Huang K.J., Wang P.Y., Chang I.Y., Wang H.I., Ma K.T., Wang C.Y., Huang X.R. (2020). SNAP29 mediates the assembly of histidine-induced CTP synthase filaments in proximity to the cytokeratin network. J. Cell. Sci..

[63] Savage C.R., Weinfeld H. (1970). Purification and properties of mammalian liver cytidine triphosphate synthetase. J. Biol. Chem..

[64] Levitzki A., Koshland D.E. (1972). Role of an allosteric effector. Guanosine triphosphate activation in cytosine triphosphate synthetase. Biochemistry.

[65] Kizaki H., Ohsaka F., Sakurada T. (1982). Role of GTP in CTP synthetase from Ehrlich ascites tumor cells. Biochem. Biophys. Res. Commun..

[66] Zhou X., Guo C.J., Chang C.C., Zhong J., Hu H.H., Lu G.M., Liu J.L. (2021). Structural basis for ligand binding modes of CTP synthase. Proc. Natl. Acad. Sci. USA.

[67] Lauritsen I., Willemoës M., Jensen K.F., Johansson E., Harris P. (2011). Structure of the dimeric form of CTP synthase from *Sulfolobus solfataricus*. Acta Crystallogr..

[68] Kursula P., Flodin S., Ehn M., Hammarstrom M., Schuler H., Nordlund P. (2006). Structure of the synthetase domain of human CTP synthetase, a target for anticancer therapy. Acta Crystallograph. Sect. F Struct. Biol. Cryst. Commun..

[69] Zhou X., Guo C.J., Hu H.H., Zhong J., Sun Q., Liu D., Zhou S., Chang C.C., Liu J.L. (2019). Drosophila CTP synthase can form distinct substrate- and product-bound filaments. J. Genet. Genom..

[70] Robertson J.G., Villafranca J.J. (1993). Characterization of metal ion activation and inhibition of CTP synthetase. Biochemistry.

[71] MacDonnell J.E., Lunn F.A., Bearne S.L. (2004). Inhibition of *E. coli* CTP synthase by the “positive” allosteric effector GTP. Biochim. Biophys. Acta.

[72] Lunn F.A., MacDonnell J.E., Bearne S.L. (2008). Structural requirements for the activation of *Escherichia coli* CTP synthase by the allosteric effector GTP are stringent, but requirements for inhibition are lax. J. Biol. Chem..

[73] Wadskov-Hansen S.L., Willemoës M., Martinussen J., Hammer K., Neuhard J., Larsen S. (2001). Cloning and verification of the *Lactococcus lactis* pyrG gene and characterization of the gene product, CTP synthase. J. Biol. Chem..

[74] Nadkarni A.K., McDonough V.M., Yang W.L., Stukey J.E., Ozier-Kalogeropoulos O., Carman G.M. (1995). Differential biochemical regulation of the URA7- and URA8-encoded CTP synthetases from *Saccharomyces cerevisiae*. J. Biol. Chem..

[75] Bearne S.L., Hekmat O., MacDonnell J.E. (2001). Inhibition of *Escherichia coli* CTP synthase by glutamate γ-semialdehyde and the role of the allosteric effector GTP in glutamine hydrolysis. Biochem. J..

[76] Willemoës M. (2004). Competition between ammonia derived from internal glutamine hydrolysis and hydroxylamine present in the solution for incorporation into UTP as catalysed by *Lactococcus lactis* CTP synthase. Arch. Biochem. Biophys..

[77] Mareya S.M., Raushel F.M. (1994). A molecular wedge for triggering the amidotransferase activity of carbamoyl phosphate synthetase. Biochemistry.

[78] Miles B.W., Banzon J.A., Raushel F.M. (1998). Regulatory control of the amidotransferase domain of carbamoyl phosphate synthetase. Biochemistry.

[79] Myers R.S., Jensen J.R., Deras I.L., Smith J.L., Davisson V.J. (2003). Substrate-induced changes in the ammonia channel for imidazole glycerol phosphate synthase. Biochemistry.

[80] Roy A.C., Lunn F.A., Bearne S.L. (2010). Inhibition of CTP synthase from *Escherichia coli* by xanthines and uric acids. Bioorg. Med. Chem. Lett..

[81] Simard D., Hewitt K.A., Lunn F., Iyengar A., Bearne S.L. (2003). Limited proteolysis of *Escherichia coli* cytidine-5′-triphosphate synthase. Identification of residues required for CTP formation and GTP-dependent activation of glutamine hydrolysis. Eur. J. Biochem..

[82] Iyengar A., Bearne S.L. (2003). Aspartate 107 and leucine 109 facilitate efficient coupling of glutamine hydrolysis to CTP synthesis by *E. coli* CTP synthase. Biochem. J..

[83] Lunn F.A., Bearne S.L. (2004). Alternative substrates for wild-type and L109A *E. coli* CTP synthases. Kinetic evidence for a constricted ammonia tunnel. Eur. J. Biochem..

[84] Willemoës M., Mølgaard A., Johansson E., Martinussen J. (2005). Lid L11 of the glutamine amidotransferase domain of CTP synthase mediates allosteric GTP activation of glutaminase activity. FEBS J..

[85] Willemoës M. (2003). Thr-431 and Arg-433 are part of a conserved sequence motif of the glutamine amidotransferase domain of CTP synthases and are involved in GTP activation of the *Lactococcus lactis* enzyme. J. Biol. Chem..

[86] McCluskey G.D., Bearne S.L. (2018). “Pinching” the ammonia tunnel of CTP synthase unveils coordinated catalytic and allosteric-dependent control of ammonia passage. Biochim. Biophys. Acta Gen. Subj..

[87] Habrian C., Chandrasekhara A., Shahrvini B., Hua B., Lee J., Jesinghaus R., Barry R., Gitai Z., Kollman J., Baldwin E.P. (2016). Inhibition of *Escherichia coli* CTP synthetase by NADH and other nicotinamides and their mutual interactions with CTP and GTP. Biochemistry.

[88] Ashkenazy H., Abadi S., Martz E., Chay O., Mayrose I., Pupko T., Ben-Tal N. (2016). ConSurf 2016: An improved methodology to estimate and visualize evolutionary conservation in macromolecules. Nucleic Acids Res..

[89] Levitzki A., Stallcup W.B., Koshland D.E. (1971). Half-of-the-sites reactivity and the conformational states of cytidine triphosphate synthetase. Biochemistry.

[90] Yoon J., Cho L.H., Kim S.R., Tun W., Peng X., Pasriga R., Moon S., Hong W.J., Ji H., Jung K.H. (2021). CTP synthase is essential for early endosperm development by regulating nuclei spacing. Plant Biotechnol. J..

[91] Daumann M., Hickl D., Zimmer D., DeTar R.A., Kunz H.H., Möhlmann T. (2018). Characterization of filament-forming CTP synthases from *Arabidopsis thaliana*. Plant J..

[92] Wylie J.L., Berry J.D., McClarty G. (1996). *Chlamydia trachomatis* CTP synthetase: Molecular characterization and developmental regulation of expression. Mol. Microbiol..

[93] Yuan P., Hendriks E.F., Fernandez H.R., O’Sullivan W.J., Stewart T.S. (2005). Functional expression of the gene encoding cytidine triphosphate synthetase from *Plasmodium falciparum* which contains two novel sequences that are potential antimalarial targets. Mol. Biochem. Parasitol..

[94] Jiménez B.M., O’Sullivan W.J. (1994). CTP synthetase and enzymes of pyrimidine ribonucleotide metabolism in *Giardia intestinalis*. Int. J. Parasitol..

[95] O’Sullivan W.J., Jiminez B.M., Dai Y.P., Lee C.S. (1991). GTP activates two enzymes of pyrimidine salvage from the human intestinal parasite *Giardia intestinalis*. Adv. Exp. Med. Biol..

[96] Yang W.L., McDonough V.M., Ozier-Kalogeropoulos O., Adeline M.T., Flocco M.T., Carman G.M. (1994). Purification and characterization of CTP synthetase, the product of the URA7 gene in *Saccharomyces cerevisiae*. Biochemistry.

[97] Yang W.L., Bruno M.E., Carman G.M. (1996). Regulation of yeast CTP synthetase activity by protein kinase C. J. Biol. Chem..

[98] Yang W.L., Carman G.M. (1996). Phosphorylation and regulation of CTP synthetase from *Saccharomyces cerevisiae* by protein kinase A. J. Biol. Chem..

[99] Park T.S., Ostrander D.B., Pappas A., Carman G.M. (1999). Identification of Ser424 as the protein kinase A phosphorylation site in CTP synthetase from *Saccharomyces cerevisiae*. Biochemistry.

[100] Kassel K.M., Au D.R., Higgins M.J., Hines M., Graves L.M. (2010). Regulation of human cytidine triphosphate synthetase 2 by phosphorylation. J. Biol. Chem..

[101] McPartland R.P., Weinfeld H. (1979). Cooperative effects of CTP on calf liver CTP synthetase. J. Biol. Chem..

[102] Weinfeld H., Savage C.R., McPartland R.P. (1978). CTP synthetase of bovine calf liver. Methods Enzymol..

[103] Aronow B., Ullman B. (1987). In situ regulation of mammalian CTP synthetase by allosteric inhibition. J. Biol. Chem..

[104] Kizaki H., Sakurada T., Weber G. (1981). Purification and properties of CTP synthetase from Ehrlich ascites tumor cells. Biochim. Biophys. Acta.

[105] Kizaki H., Ohsaka F., Sakurada T. (1985). CTP synthetase from Ehrlich ascites tumor cells. Subunit stoichiometry and regulation of activity. Biochim. Biophys. Acta.

[106] Ozier-Kalogeropoulos O., Fasiolo F., Adeline M.T., Collin J., Lacroute F. (1991). Cloning, sequencing and characterization of the *Saccharomyces cerevisiae* URA7 gene encoding CTP synthetase. Mol. Gen. Genet..

[107] Ozier-Kalogeropoulos O., Adeline M.T., Yang W.L., Carman G.M., Lacroute F. (1994). Use of synthetic lethal mutants to clone and characterize a novel CTP synthetase gene in *Saccharomyces cerevisiae*. Mol. Gen. Genet..

[108] Yang W.L., Carman G.M. (1995). Phosphorylation of CTP synthetase from *Saccharomyces cerevisiae* by protein kinase C. J. Biol. Chem..

[109] Van Kuilenburg A.B., Meinsma R., Vreken P., Waterham H.R., van Gennip A.H. (2000). Isoforms of human CTP synthetase. Adv. Exp. Med. Biol..

[110] Van Kuilenburg A.B.P., Meinsma R., Vreken P., Waterham H.R., van Gennip A.H. (2000). Identifcation of a cDNA encoding an isoform of human CTP synthetase. Biochim. Biophys. Acta.

[111] Van Kuilenburg A.B., Elzinga L., van Gennip A.H. (1998). Kinetic properties of CTP synthetase from HL-60 cells. Adv. Exp. Med. Biol..

[112] Traut T.W. (1994). Physiological concentrations of purines and pyrimidines. Mol. Cell. Biochem..

[113] Liu J.L. (2010). Intracellular compartmentation of CTP synthase in *Drosophila*. J. Genet. Genom..

[114] Zhang Y., Liu J., Liu J.-L. (2020). The atlas of cytoophidia in *Drosophila* larvae. J. Genet. Genom..

[115] Noree C., Sato B.K., Broyer R.M., Wilhelm J.E. (2010). Identification of novel filament-forming proteins in *Saccharomyces cerevisiae* and *Drosophila melanogaster*. J. Cell Biol..

[116] Zhang J., Hulme L., Liu J.-L. (2014). Asymmetric inheritance of cytoophidia in *Schizosaccharomyces pombe*. Biol. Open.

[117] Zhou S., Xiang H., Liu J.-L. (2020). CTP synthase forms cytoophidia in archaea. J. Genet. Genom..

[118] Chang C.C., Keppeke G.D., Antos C.L., Peng M., Andrade L.E.C., Sung L.Y., Liu J.-L. (2021). CTPS forms the cytoophidium in zebrafish. Exp. Cell Res..

[119] Chen K., Zhang J., Tastan Ö.Y., Deussen Z.A., Siswick M.Y., Liu J.L. (2011). Glutamine analogs promote cytoophidium assembly in human and *Drosophila* cells. J. Genet. Genom..

[120] Peng M., Chang C.C., Liu J.L., Sung L.Y. (2021). CTPS and IMPDH form cytoophidia in developmental thymocytes. Exp. Cell Res..

[121] Hansen J.M., Horowitz A., Lynch E.M., Farrell D.P., Quispe J., DiMaio F., Kollman J.M. (2021). Cryo-EM structures of CTP synthase filaments reveal mechanism of pH-sensitive assembly during budding yeast starvation. eLife.

